# First synthesis of *meso*-substituted pyrrolo[1,2-*a*]quinoxalinoporphyrins

**DOI:** 10.3762/bjoc.10.76

**Published:** 2014-04-08

**Authors:** Dileep Kumar Singh, Mahendra Nath

**Affiliations:** 1 Department of Chemistry, University of Delhi, Delhi 110 007, India

**Keywords:** Clauson–Kaas reaction, fluorescence, pyrrolo[1,2-*a*]quinoxalinoporphyrins, Pictet–Spengler reaction, synthesis

## Abstract

A synthetic protocol for the construction of new *meso*-substituted pyrrolo[1,2-*a*]quinoxalinoporphyrins is described starting from 5-(4-amino-3-nitrophenyl)-10,15,20-triphenylporphyrin. The reaction of this porphyrin with 2,5-dimethoxytetrahydrofuran, followed by the reduction of the nitro group in the presence of NiCl_2_/NaBH_4_ afforded 5-(3-amino-4-(pyrrol-1-yl)phenyl)-10,15,20-triphenylporphyrin. This triphenylporphyrin underwent a Pictet–Spengler cyclization after the reaction with various aromatic aldehydes followed by in situ KMnO_4_ oxidation to form target porphyrin analogues in good yields. The structures of all synthesized products were established on the basis of spectral data and elemental analyses.

## Introduction

Many natural porphyrins are known to play essential roles in a number of biological processes including oxygen transport [1], solar energy conservation [2–4] and photosynthesis [5]. Owing to the expanded π-conjugation system as well as good thermal stabilities, various artificial porphyrins have been prepared as promising materials for organic photonic and electronic applications [6–9]. In addition, porphyrins fused with external aromatic systems exhibit a broad range of applications in diverse areas such as molecular devices [10–13], organic light emitting diodes [14–15], near infrared dyes [16–18], hybrid solar cells [19–22], and biosensors [23–25] due to their intense optical absorptions and photoluminescence characteristics. On the other hand, compounds containing a pyrrolo[1,2-*a*]quinoxaline subunit display a wide spectrum of biological profiles as antagonists [26–27], PARP-1 inhibitors [28], anticancer agents [29–30], anti-HIV agents [31], and antimalarial agents [32–33]. These molecules are also important intermediates for the construction of 5-HT_3_ receptor agonists [34–35] and are useful as fluorescent materials for various applications [36–37].

In recent years, numerous covalent or non-covalent supra-porphyrin arrays, based on donor–acceptor architectures have been constructed for mimicking the natural photosynthetic light harvesting systems [38–40]. Additionally, a variety of biologically important functional groups were also introduced on the periphery of *meso*-substituted porphyrins to develop efficient photosensitizers for photodynamic therapy applications [41–43]. However, the porphyrins with a pyrrolo[1,2-*a*]quinoxaline moiety at the *meso*-positions have not been synthesized and their photophysical properties have not been evaluated yet. By considering the biological and fluorescent properties of these two classes of heterocycles, we envisaged to combine both porphyrin and pyrrolo[1,2-*a*]quinoxaline units in a single molecular framework to generate novel *meso*-substituted pyrrolo[1,2-a]quinoxalinoporphyrin analogues. Such hybrid molecules may prove useful for various biological studies and in the development of new photodynamic agents. Therefore, in continuation of our efforts to develop simple and efficient methods [44–48] for the synthesis of diverse porphyrin derivatives from *meso*-tetraarylporphyrins, we wish to report herein the first synthesis and spectroscopic properties of a novel series of *meso*-substituted pyrrolo[1,2-*a*]quinoxalinoporphyrins.

## Results and Discussion

**Scheme 1:**
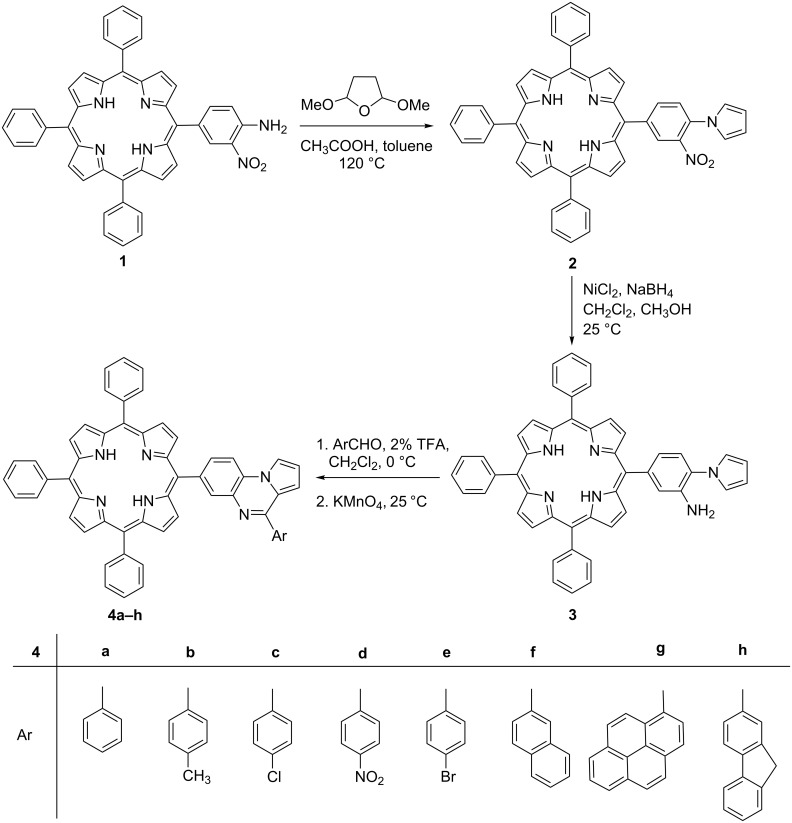
Synthesis of pyrrolo[1,2-*a*]quinoxalinoporphyrins (**4a**–**h**).

The synthetic strategy for targeted *meso*-substituted pyrrolo[1,2-*a*]quinoxalinoporphyrins (**4a–h**) is depicted in Scheme 1. At first, 5-(4-amino-3-nitrophenyl)-10,15,20-triphenylporphyrin (**1**) was synthesized from 5,10,15,20-tetraphenylporphyrin (TPP) after a series of reactions [46,49] in five steps. The Clauson–Kaas reaction of porphyrin (**1**) with 2,5-dimethoxytetrahydrofuran in toluene/acetic acid mixture afforded novel 5-(3-nitro-4-(pyrrol-1-yl)phenyl)-10,15,20-triphenylporphyrin (**2**) in 89% yield. The reduction of nitroporphyrin **2** was initially carried out by using Sn/HCl, SnCl_2_·2H_2_O/HCl, and Pd/C–NaBH_4_ as reducing agents but the reaction was found to be sluggish and provided an inseparable mixture of products. Instead, nitroporphyrin **2** was successfully reduced to 5-(3-amino-4-(pyrrol-1-yl)phenyl)-10,15,20-triphenylporphyrin (**3**) in the presence of nickel boride, generated in situ by the reaction of NiCl_2_ and NaBH_4_ in a CH_2_Cl_2_/MeOH mixture at 25 °C. Finally, the synthesis of novel *meso*-substituted pyrrolo[1,2-*a*]quinoxalinoporphyrins (**4a–h**) began via the Pictet–Spengler cyclization reaction [50–51] of 5-(3-amino-4-(pyrrol-1-yl)phenyl)-10,15,20-triphenylporphyrin (**3**) with various aromatic aldehydes by using 2% TFA in dichloromethane as an acidic catalyst at 0 °C for 5 minutes, followed by aromatization in the presence of KMnO_4_ at room temperature (Scheme 1).

**Scheme 2:**
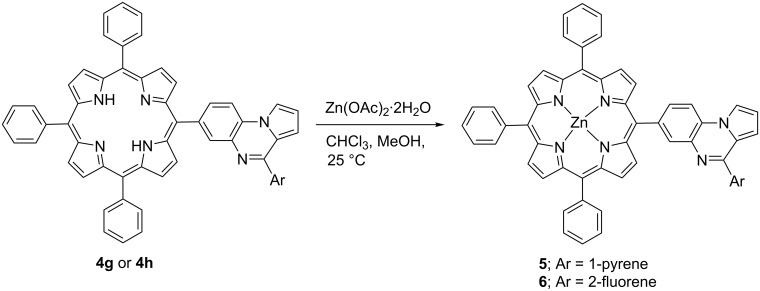
Synthesis of zinc(II) pyrrolo[1,2-*a*]quinoxalinoporphyrins **5** and **6**.

The target products were purified by column chromatography over neutral alumina and obtained in 60–76% isolated yields. Furthermore, the π electron-rich free-base porphyrin dyads (**4g** and **4h**) were converted to the corresponding zinc(II) porphyrins (**5** and **6**) in 84 and 87% yields, respectively, after the treatment with Zn(OAc)_2_·2H_2_O in CHCl_3_/MeOH mixture for 30 minutes at room temperature (Scheme 2).

All synthesized porphyrins (**2**, **3**, **4a–h, 5** and **6**) were characterized on the basis of NMR, IR, UV–vis and mass spectral data in addition to elemental analysis. The proton NMR of newly prepared free-base *meso*-substituted pyrrolo[1,2-*a*]quinoxalinoporphyrins (**4a–h**) showed a characteristic singlet around δ −2.7 ppm for two NH protons of the porphyrin core. The β-pyrrolic protons of the porphyrin ring appeared in the downfield region between δ 8.85–9.01 ppm. A characteristic doublet at δ 8.9 and a double doublet at δ 8.3 ppm were assigned to the C-2 and C-6 protons of the *meso*-phenyl ring fused with the pyrroloquinoxaline moiety. The C-5 proton was found to be merged with nine other *meso*-phenyl protons and appeared as a multiplet between δ 7.75–7.77 ppm. The remaining six *meso*-phenyl protons appeared as a multiplet between δ 8.20–8.25 ppm along with a pyrrolic C-1′ proton. In the case of porphyrins (**4a–f**), the two pyrrolic C-2′ and C-3′ protons of the pyrroloquinoxaline ring appeared as a double doublet at around δ 7.06 ppm and a doublet at around δ 7.19 ppm, respectively. The ^1^H NMR spectrum of porphyrin **4g** displayed these pyrrolic C-2′ and C-3′ protons as a double doublet at δ 7.01 ppm and a doublet at around δ 6.74 ppm, whereas these pyrrolic protons appeared as multiplets between δ 7.09–7.24 ppm in the case of porphyrin **4h**. In addition, porphyrin **4h** and **6** showed a characteristic singlet for the CH_2_ protons of the fluorenyl moiety at δ 4.0 and 3.9 ppm, respectively. The IR spectra of all the free-base pyrrolo[1,2-*a*]quinoxalinoporphyrins showed a peak between 3317–3318 cm^−1^ due to the NH bond stretching. The structures of porphyrins (**2**, **3**, **4a–h, 5** and **6**) were further supported by mass spectral analysis, which revealed the molecular ion peak to be [M + H]^+^. The electronic absorption and emission data of all the synthesized compounds are presented in Table 1.

**Table 1:** Electronic absorption and emission data of porphyrins (**2, 3, 4a**–**h**, **5** and **6**).

Compound	Absorption^a^ λ_max_, nm (ε × 10^−4^, M^−1^ cm^−1^)	Fluorescence^a,b^ (λ_em_/nm)

**2**	421 (56.24), 517 (2.82), 551 (1.26), 593 (0.41), 647 (0.54)	651, 717
**3**	421 (45.66), 517 (2.56), 550 (1.28), 597 (0.31), 647 (0.80)	653, 717
**4a**	422 (39.00), 517 (2.93), 550 (1.84), 597 (0.33), 647 (0.97)	653, 717
**4b**	422 (58.51), 517 (3.11), 551 (1.63), 594 (0.37), 647 (0.71)	652, 717
**4c**	422 (51.51), 517 (2.36), 552 (1.16), 594 (0.41), 648 (0.59)	652, 716
**4d**	422 (57.74), 517 (3.49), 552 (1.81), 596 (0.30), 648 (0.76)	652, 715
**4e**	422 (61.39), 517 (3.05), 552 (1.53), 597 (0.19), 648 (0.68)	652, 716
**4f**	423 (63.00), 517 (3.50), 551 (1.91), 597 (0.34), 647 (0.97)	652, 717
**4g**	422 (59.97), 517 (3.32), 552 (1.82), 596 (0.32), 648 (0.87)	652, 717
**4h**	423 (73.28), 517 (3.90), 552 (2.07), 596 (0.38), 648 (0.96)	652, 717
**5**	425 (104.90), 554 (3.80), 594 (1.00)	605, 652
**6**	425 (117.50), 553 (4.20), 594 (1.20)	606, 654

^a^Absorption and emission data were taken for CHCl_3_ solutions of porphyrins at 298 K. ^b^The excitation wavelength for emission data is 420 nm.

**Figure 1:**
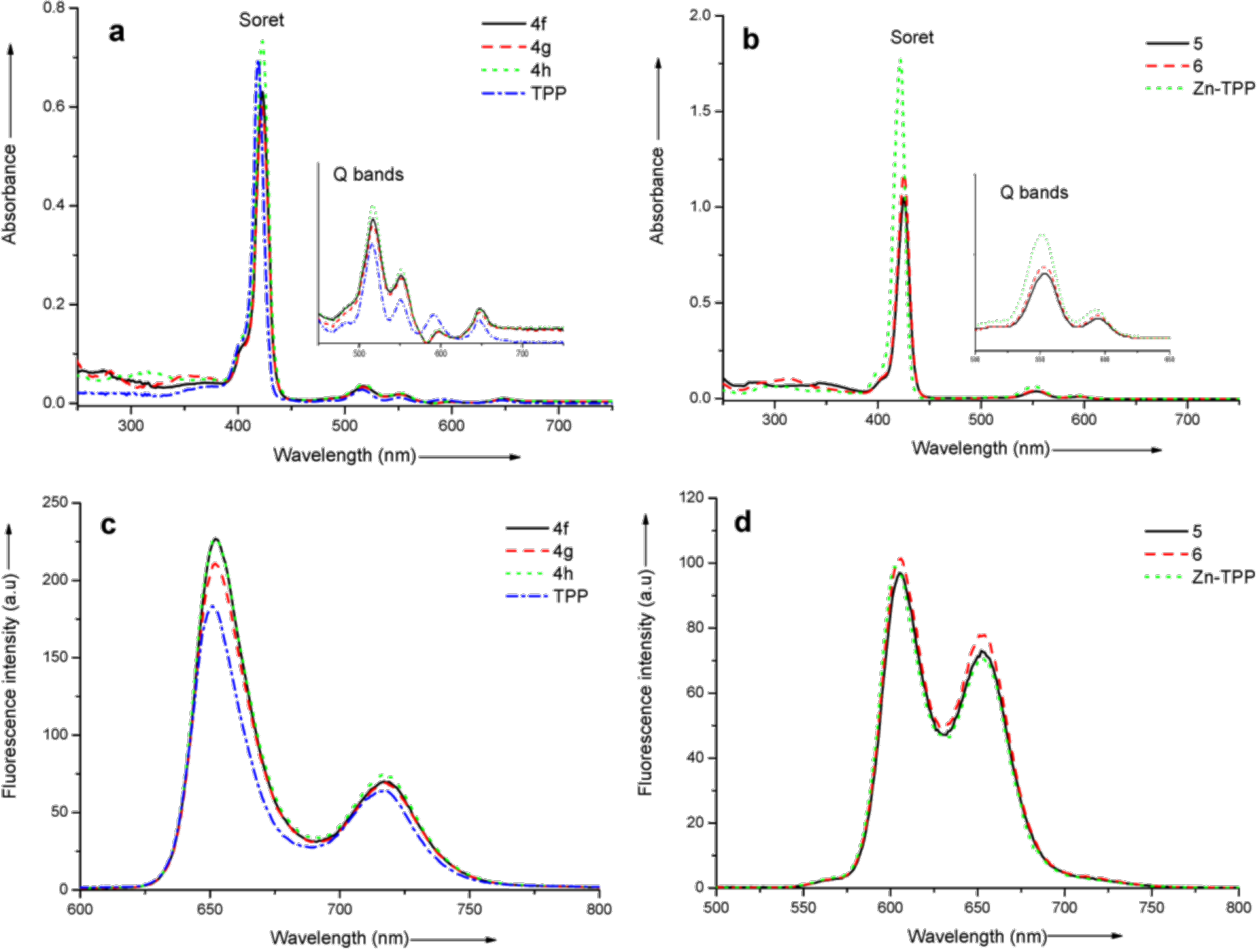
(a) Electronic absorption spectra of free-base porphyrins **4f**, **4g**, **4h** and TPP in CHCl_3_ (1 × 10^−6^ mol L^−1^) at 298 K. (b) Electronic absorption spectra of zinc porphyrins (**5**, **6** and Zn–TPP) in CHCl_3_ (2 × 10^−6^ mol L^−1^) at 298 K. The inset in both (a) and (b) shows the Q bands. (c) Fluorescence spectra of porphyrins **4f**, **4g**, **4h** and TPP in CHCl_3_ (1 × 10^−6^ mol L^−1^) at 298 K, λ_ex_ = 420 nm. (d) Fluorescence spectra of zinc porphyrins (**5**, **6**, and Zn–TPP) in CHCl_3_ (2 × 10^−6^ mol L^−1^) at 298 K, λ_ex_ = 420 nm.

The UV–vis spectra of newly prepared *meso-*substituted pyrrolo[1,2-*a*]quinoxalinoporphyrins (**4a**–**h**) in chloroform exhibited a typical intense Soret band at ~422 nm and four weaker Q bands at ~517, 552, 596 and 647 nm. In contrast, the zinc(II) pyrrolo[1,2-*a*]quinoxalinoporphyrin analogues **5** and **6** showed an intense Soret band at ~425 nm and two weaker Q bands at ~553 and 594 nm. In comparison to the TPP and Zn–TPP, the UV–vis spectra of free-base porphyrins **4a**–**h** and zinc porphyrins (**5** and **6**) were found to be red-sifted by 3 to 4 nm. The electronic absorption spectra of selected free-base porphyrins (**4f**, **4g**, **4h** and TPP) and zinc(II) porphyrins (**5**, **6** and Zn–TPP) are shown in Figure 1a,b. Besides the Soret and Q bands in porphyrins **4g**, **4h**, **5** and **6**, an additional absorption peak originates at 280 and 320 nm due to the presence of pyrene and fluorene units, respectively. Thus, the electronic absorption spectra of these compounds demonstrated the features of both porphyrin and pyrene or fluorene subunits and suggest that there is no significant interaction between the attached chromophore and the porphyrin ring in the ground state.

The fluorescence spectra of porphyrins **4f**, **4g**, **4h**, **5** and **6** were recorded in CHCl_3_ at the excitation wavelength of 420 nm and are shown in Figure 1c,d. The free-base pyrrolo[1,2-*a*]quinoxalinoporphyrins **4f**, **4g** and **4h** displayed an emission band and a weak shoulder at ~652 and ~717 nm. These emission bands are found to be slightly intense in comparison to the TPP (Figure 1c). Similarly, the zinc(II) pyrrolo[1,2-*a*]quinoxalinoporphyrins **5** and **6** showed two fluorescence bands at ~605 and ~652 nm, which are also found to be slightly intense when compared to the emission bands of Zn–TPP (Figure 1d).

## Conclusion

In summary, the synthesis of two new porphyrin building blocks, 5-(3-nitro-4-(pyrrol-1-yl)phenyl)-10,15,20-triphenylporphyrin (**2**) and 5-(3-amino-4-(pyrrol-1-yl)phenyl)-10,15,20-triphenylporphyrin (**3**), has been accomplished in good yields. The porphyrin **3** was successfully utilized as starting material for the construction of a novel series of *meso*-substituted pyrrolo[1,2-*a*]quinoxalinoporphyrins in 60–76% yields via TFA-catalyzed Pictet–Spengler cyclization with aromatic aldehydes followed by in situ oxidation in the presence of KMnO_4_. These porphyrin architectures may be useful as potential candidates for various biological evaluations.

## Supporting Information

File 1 Experimental details and characterization data.

File 2 ^1^H and ^13^C NMR spectra of newly synthesized compounds.
