# Supplementary material for: First synthesis of meso-substituted pyrrolo[1,2-a]quinoxalinoporphyrins
Source: Beilstein J Org Chem. 2014 Apr 8;10:808–13. doi: 10.3762/bjoc.10.76 (PMC4001320; doi:10.3762/bjoc.10.76)
Supplement: File 1 — Experimental details and characterization data. [file Beilstein_J_Org_Chem-10-808-s001.pdf]

Supporting Information File 1

for

First synthesis of *meso*-substituted pyrrolo[1,2-*a*]quinoxalinoporphyrins

Dileep Kumar Singh and Mahendra Nath*

Address: Department of Chemistry, University of Delhi, Delhi 110 007, India

Email: Mahendra Nath* - mnath@chemistry.du.ac.in

*Corresponding author

Experimental details and characterization data

General:

The reagents and solvents required for the present study were purchased from Sigma-Aldrich and Merck. The progress of the reactions was monitored by thin-layer chromatography (TLC) on silica gel 60 F_{254} (pre-coated aluminium sheets). The products were purified by column chromatography using either activated neutral aluminium oxide (Brokmann grade I–II, Merck) or silica gel (60–120 mesh). ^{1}H NMR (400 MHz) and ^{13}C NMR (100 MHz) spectra were recorded in $CDCl_3$ on Jeol ECX 400P (400 MHz) NMR spectrometer. Chemical shifts are reported in parts per million (ppm) on the δ scale relative to an internal standard. Coupling constants *J* are reported in Hertz (Hz). Elemental analyses for all the synthesized products were performed on Elementar Analysensysteme GmbH VarioEL elemental analyzer. Infrared spectra were recorded on a Perkin Elmer IR spectrometer and absorption maxima (ν_{max}) are given in cm^{-1}. The mass spectra (ESI–MS) were recorded on a Thermo Finnigan LCQ Advantage max ion trap mass spectrometer. Electronic absorption and

emission spectra were recorded on an Analytik Jena's Specord 250 UV–vis spectrophotometer and a Varian Cary Eclipse fluorescence spectrophotometer, respectively by using spectroscopic grade $CHCl_3$ as a solvent. The melting points of newly prepared porphyrins (**2**, **3**, **4a–h**, **5** and **6**) were determined in open capillary tubes on an Büchi M-560 melting point apparatus and are uncorrected.

Synthesis of 5-(3-nitro-4-(pyrrol-1-yl)phenyl)-10,15,20-triphenylporphyrin (2).

5-(4-Amino-3-nitrophenyl)-10,15,20-triphenylporphyrin (50 mg, 0.074 mmol) was dissolved in a mixture of acetic acid (10 mL) and toluene (2 mL). To this solution, 2,5-dimethoxytetrahydrofuran (19 µL, 0.148 mmol) was added and reaction mixture was stirred at 120 °C for an hour. After completion of the reaction as indicated by TLC, the reaction mixture was allowed to cool to room temperature. The solvent was evaporated under reduced pressure and thus, the residue obtained was dissolved in chloroform (50 mL). The solution was washed thoroughly with water (3 × 30 mL), dried over anhydrous sodium sulfate and evaporated to dryness. The crude product was purified on a silica gel column using 80% chloroform in hexane as eluent.

Purple solid; yield: (48 mg, 89%); mp >300 °C; UV λ_{max} ($\varepsilon \times 10^{-4}$, M^{-1} cm^{-1}): 421 (56.24), 517 (2.82), 551 (1.26), 593 (0.41) and 647 (0.54) nm; λ_{Em} ($CHCl_3$; λ_{Ex} 420 nm): 651, 717 nm; IR (film) ν_{max}/cm^{-1}: 3320, 1537, 1476, 1350, 1216, 1088, 969, 801, 753, 730, 702; ^1H NMR (400 MHz, $CDCl_3$) δ: 8.94 (2H, d, J = 4.39 Hz, *β*-pyrrolic H), 8.88 (4H, s, *β*-pyrrolic H), 8.85 (2H, d, J = 4.39 Hz, *β*-pyrrolic H), 8.71(1H, d, J = 1.46 Hz, *meso*-ArH), 8.47 (1H, dd, J_1 = 8.05, J_2 = 2.20 Hz, *meso*-ArH), 8.24-8.22 (6H, m, *meso*-ArH), 7.85 (1H, d, J = 8.05 Hz, *meso*-ArH), 7.81-7.77 (9H, m, *meso*-ArH), 7.14-7.13 (2H, m, pyrrolic H), 6.55-6.54 (2H, m, pyrrolic H), -2.76 (2H, s, internal NH); ^{13}C NMR (100 MHz, $CDCl_3$) δ: 143.52, 142.08, 141.87, 141.83, 138.31, 134.54, 133.56, 131.44, 129.76, 127.90, 126.74, 125.78, 121.50, 121.17, 120.76,

115.21, 111.54; ESI-MS: m/z = 725.4 $(M+H)^+$; Anal. Calcd for $C_{48}H_{32}N_6O_2 \cdot H_2O$: C, 77.61; H, 4.61; N, 11.31; found: C, 77.72; H, 4.67; N; 11.26.

Synthesis of 5-(3-amino-4-(pyrrol-1-yl)phenyl)-10,15,20-triphenylporphyrin (3).

To a solution of 5-(3-nitro-4-(pyrrol-1-yl)phenyl)-10,15,20-triphenylporphyrin (50 mg, 0.068 mmol) in a mixture of dry dichloromethane (3 mL) and methanol (6 mL), anhydrous $NiCl_2$ (54 mg, 0.416 mmol) was added with stirring at room temperature. $NaBH_4$ (16 mg, 0.422 mmol) was added in portions and the reaction mixture was stirred at room temperature for 5 minutes. After completion of the reaction, the mixture was filtered through celite. The filtrate was evaporated under reduced pressure and thus, the residue obtained was dissolved in chloroform (50 mL). The resulting solution was washed with water (3 × 30 mL), dried over anhydrous Na_2SO_4 and evaporated to dryness. The crude product was purified on silica gel column using chloroform as eluent.

Purple solid; yield: (31 mg, 64%); mp >300°C; UV λ_{max} ($\varepsilon \times 10^{-4}$, M^{-1} cm^{-1}): 421 (45.66), 517 (2.56), 550 (1.28), 597 (0.31) and 647 (0.80) nm; λ_{Em} ($CHCl_3$; λ_{Ex} 420 nm): 653, 717 nm; IR (film) ν_{max}/cm^{-1}: 3490, 3382, 3319, 1597, 1474, 1350, 1218, 1071, 971, 800, 728, 751, 728, 700; ^1H NMR (400 MHz, $CDCl_3$) δ: 9.01 (2H, d, J = 5.13 Hz, β-pyrrolic H), 8.87 (2H, d, J = 4.39 Hz, β-pyrrolic H), 8.85 (4H, s, β-pyrrolic H), 8.23-8.21 (6H, m, *meso*-ArH), 7.79-7.73 (9H, m, *meso*-ArH), 7.67-7.66 (1H, m, *meso*-ArH), 7.64 (1H, d, J = 1.46 Hz, *meso*-ArH), 7.50 (1H, d, J = 7.32 Hz, *meso*-ArH), 7.19-7.18 (2H, m, pyrrolic H), 6.52-6.51 (2H, m, pyrrolic H), 4.01 (2H, brs, NH_2), -2.77 (2H, s, internal NH); ^{13}C NMR (100 MHz, $CDCl_3$) δ: 142.41, 142.13, 142.01, 139.95, 134.56, 131.01, 127.72, 127.18, 126.68, 125.61, 125.03, 122.87, 121.92, 120.25, 120.16, 119.31, 109.74; ESI-MS: m/z = 695.5 $(M+H)^+$; Anal. Calcd for $C_{48}H_{34}N_6$: C, 82.97; H, 4.93; N, 12.10; found: C, 83.02; H, 4.78; N, 11.87.

General procedure for the synthesis of free-base pyrrolo[1,2-a]quinoxalinoporphyrins (4a–h).

To a solution of 5-(3-amino-4-(pyrrol-1-yl)phenyl)-10,15,20-triphenylporphyrin (30 mg, 0.043 mmol) and arylaldehyde (0.064 mmol) in dry CH_2Cl_2 (2 mL), 2% TFA in CH_2Cl_2 (0.5 mL) was added. The reaction mixture was stirred at 0 °C for 5 minutes and $KMnO_4$ (20 mg 0.126 mmol) was added. Then, the reaction mixture was further stirred at room temperature for additional 30 minutes. The progress of the reaction was monitored by TLC. After completion of the reaction, the mixture was diluted with water (50 mL) and the product was extracted with chloroform (50 mL). The organic layer was washed with water (3 × 50 mL), dried over anhydrous sodium sulfate and evaporated under reduced pressure. The crude product obtained was purified on a neutral alumina column by using 80% chloroform in hexane as eluent.

5-[4-(Phenyl)pyrrolo[1,2-*a*]quinoxalin-7-yl]-10,15,20-triphenylporphyrin (4a). Purple solid; Yield: (24 mg, 71%); mp >300°C; UV λ_{max} ($\varepsilon \times 10^{-4}$, M^{-1} cm^{-1}): 422 (39.00), 517 (2.93), 550 (1.84), 597 (0.33) and 647 (0.97) nm; λ_{Em} ($CHCl_3$; λ_{Ex} 420 nm): 653, 717 nm; IR (film) ν_{max}/cm^{-1}: 3317,1601, 1473, 1366, 1218, 1080, 972, 800, 750, 731, 699; ^1H NMR (400 MHz, $CDCl_3$) δ: 8.94 (1H, d, *J* = 1.46 Hz, *meso*-ArH), 8.92 (2H, d, *J* = 5.13 Hz, *β*-pyrrolic H), 8.87-8.83 (6H, m, *β*-pyrrolic H), 8.37-8.34 (1H, dd, J_1 = 8.42, J_2 = 1.46 Hz, *meso*-ArH), 8.26-8.20 (7H, m, pyrrolic H-1′ and *meso*-ArH), 8.14-8.12 (2H, m, ArH), 7.77-7.75 (10H, m, *meso*-ArH), 7.58-7.56 (3H, m, ArH), 7.19 (1H, d, *J* = 2.93 Hz, pyrrolic H-3′), 7.06 (1H, dd, *J* = 2.93, 2.20 Hz, pyrrolic H-2′), -2.72 (2H, s, internal NH); ^{13}C NMR (100 MHz, $CDCl_3$) δ: 155.53, 142.11, 139.33, 139.27, 138.47, 135.60, 134.89, 134.56, 133.68, 131.13, 129.96, 128.70, 128.64, 127.71, 126.86, 126.67, 125.68, 120.32, 120.25, 118.71, 115.02, 114.44, 114.07, 111.84, 109.20; ESI-MS: m/z = 781.5 $(M+H)^+$; Anal. Calcd for $C_{55}H_{36}N_6 \cdot H_2O$: C, 82.68; H, 4.79; N, 10.52; found: C, 82.62; H, 4.98; N, 10.28.

5-[4-(4-Methylphenyl)pyrrolo[1,2-*a*]quinoxalin-7-yl]-10,15,20-triphenyl-porphyrin (4b).

Purple solid; yield: (24 mg, 70%); mp >300°C; UV λ_{max} ($\varepsilon\times10^{-4}$, M^{-1} cm^{-1}): 422 (58.51), 517 (3.11), 551 (1.63), 594 (0.37) and 647 (0.71) nm; λ_{Em} ($CHCl_3$; λ_{Ex} 420 nm): 652, 717 nm; IR (film) ν_{max}/cm^{-1}: 3317, 1624, 1473, 1367, 1217, 1001, 971, 801, 753, 730, 701 ; ^{1}H NMR (400 MHz, $CDCl_3$) δ: 8.93-8.91 (3H, m, *meso*-ArH and *β*-pyrrolic H), 8.86-8.84 (6H, m, *β*-pyrrolic H), 8.34 (1H, dd, J_1 = 8.05, J_2 = 2.20 Hz, *meso*-ArH), 8.26-8.21 (7H, m, pyrrolic H-1′ and *meso*-ArH), 8.03 (2H, d, *J* = 8.05 Hz, ArH), 7.76-7.75 (10H, m, *meso*-ArH), 7.37 (2H, d, *J* = 8.05 Hz, ArH), 7.18 (1H, d, *J* = 3.66 Hz, pyrrolic H-3′), 7.05 (1H, dd, *J* = 2.93, 2.20 Hz, pyrrolic H-2′), 2.46 (3H, s, CH_3), -2.73 (2H, s, internal NH); ^{13}C NMR (100 MHz, $CDCl_3$) δ: 155.48, 142.12, 140.11, 139.26, 135.69, 135.55, 134.95, 134.56, 133.52, 131.23, 129.70, 129.53, 129.32, 128.63, 127.70, 126.85, 126.67, 125.69, 120.31, 120.25, 118.79, 114.90, 114.37, 111.81, 109.16, 21.48; ESI-MS: m/z = 795.6 $(M+H)^+$; Anal. Calcd for $C_{56}H_{38}N_6\cdot H_2O$: C, 82.73; H, 4.96; N, 10.34; found: C, 82.52; H, 5.16; N, 10.12.

5-[4-(4-Chlorophenyl)pyrrolo[1,2-*a*]quinoxalin-7-yl]-10,15,20-triphenyl-porphyrin (4c).

Purple solid; yield: (23 mg, 65%); mp >300°C; UV λ_{max} ($\varepsilon\times10^{-4}$, M^{-1} cm^{-1}): 422 (51.51), 517 (2.36), 552 (1.16), 594 (0.41) nm; λ_{Em} ($CHCl_3$; λ_{Ex} 420 nm): 652, 716 nm; IR (film) ν_{max}/cm^{-1}: 3318,1613, 1473, 1366, 1215, 1091, 971, 800, 752, 731, 700; ^{1}H NMR (400 MHz, $CDCl_3$) δ: 8.91 (1H, d, *J* = 1.46 Hz, *meso*-ArH), 8.90 (2H, d, *J* = 5.13 Hz, *β*-pyrrolic H), 8.86-8.85 (6H, m, *β*-pyrrolic H), 8.36 (1H, dd, J_1 = 8.05, J_2 = 1.46 Hz, *meso*-ArH), 8.26-8.19 (7H, m, pyrrolic H-1′ and *meso*-ArH), 8.08 (2H, d, *J* = 8.79 Hz, ArH), 7.79-7.73 (10H, m, *meso*-ArH), 7.54 (2H, d, *J* = 8.79 Hz, ArH), 7.14 (1H, d, *J* = 4.39 Hz, pyrrolic H-3′), 7.07 (1H, dd, *J* = 2.93, 2.20 Hz, pyrrolic H-2′), -2.72 (2H, s, internal NH), ^{13}C NMR (100 MHz, $CDCl_3$) δ: 154.19, 142.09, 139.47, 136.90, 136.04, 135.58, 134.76, 134.56, 133.83, 130.89, 130.04,

128.88, 128.10, 127.72, 126.82, 126.68, 125.69, 125.35, 120.39, 120.28 , 118.56, 115.19, 114.57, 111.87, 108.94; ESI-MS: m/z = 815.5 $(M+H)^+$; Anal. Calcd for $C_{55}H_{35}ClN_6$: C, 81.02; H, 4.33; N, 10.31; found: C, 80.92; H, 4.23; N, 10.46.

5-[4-(4-Nitrophenyl)pyrrolo[1,2-*a*]quinoxalin-7-yl]-10,15,20-triphenylporphyrin (4d).

Purple solid; yield: (27 mg, 76%); mp >300°C; UV λ_{max} ($\varepsilon\times10^{-4}$, M^{-1} cm^{-1}): 422 (57.74), 517 (3.49), 552 (1.81), 596 (0.30) and 648 (0.76) nm; λ_{Em} ($CHCl_3$; λ_{Ex} 420 nm): 652, 715 nm; IR (film) ν_{max}/cm^{-1}: 3318, 1598, 1474, 1345, 1222, 1099, 972, 800, 751, 730, 701; ^1H NMR (400 MHz, $CDCl_3$) δ: 8.93 (1H, d, *J* = 1.46 Hz, *meso*-ArH), 8.89-8.86 (8H, m, *β*-pyrrolic H), 8.43-8.39 (3H, m, ArH and *meso*-ArH), 8.32-8.28 (3H, m, ArH and pyrrolic H-1′), 8.23-8.22 (6H, m, *meso*-ArH), 7.77-7.75 (10H, m, *meso*-ArH), 7.13 (1H, d, *J* = 2.93 Hz, pyrrolic H-3′), 7.10 (1H, dd, *J* = 2.93, 2.20 Hz, pyrrolic H-2′), -2.72 (2H, s, internal NH); ^{13}C NMR (100 MHz, $CDCl_3$) δ: 152.87, 148.63, 144.39, 142.04, 139.72, 135.73, 134.56, 134.36, 131.19, 130.63, 130.16, 129.66, 127.75, 126.82, 126.69, 125.05, 123.99, 123.82, 120.49, 120.34, 118.23, 115.51, 114.87, 111.94, 108.71; ESI-MS: m/z = 826.5 $(M+H)^+$; Anal. Calcd for $C_{55}H_{35}N_7O_2$: C, 79.98; H, 4.27; N, 11.87; found: C, 80.02; H, 4.34; N, 11.68.

5-[4-(4-Bromophenyl)pyrrolo[1,2-*a*]quinoxalin-7-yl]-10,15,20-triphenyl-porphyrin (4e).

Purple solid; yield: (25 mg, 67%); mp >300°C; UV λ_{max} ($\varepsilon\times10^{-4}$, M^{-1} cm^{-1}): 422 (61.39), 517 (3.05), 552 (1.53), 597 (0.19) and 648 (0.68) nm; λ_{Em} ($CHCl_3$; λ_{Ex} 420 nm): 652, 716 nm; IR (film) ν_{max}/cm^{-1}: 3320, 1590, 1474, 1367, 1218, 1070, 972, 801, 752, 730, 701; ^1H NMR (400 MHz, $CDCl_3$) δ: 8.91 (1H, d, *J* = 1.46, *meso*-ArH), 8.90 (2H, d, *J* = 5.13 Hz, *β*-pyrrolic H), 8.87-8.85 (6H, m, *β*-pyrrolic H), 8.36 (1H, dd, J_1 = 8.05, J_2 = 1.46 Hz, *meso*-ArH), 8.26-8.19 (7H, m, pyrrolic H-1′ and *meso*-ArH), 8.01 (2H, d, *J* = 8.79 Hz, ArH), 7.77-7.75 (10H, m, *meso*-ArH), 7.70 (2H, d, *J* = 8.79 Hz, ArH), 7.14 (1H, d, *J* = 3.66 Hz, pyrrolic H-3′), 7.07

(1H, dd, J = 2.93, 2.20 Hz, pyrrolic H-2′), -2.72 (2H, s, internal NH), ^{13}C NMR (100 MHz, $CDCl_3$) δ: 154.22, 142.08, 139.46, 137.34, 135.57, 134.75, 134.55, 133.84, 132.38, 131.83, 131.13, 130.90, 130.27, 127.72, 126.80, 126.68, 125.28, 124.36, 120.39, 120.28, 118.54, 115.18, 114.57, 111.85, 108.92; ESI-MS: m/z = 859.5 $(M+H)^+$; Anal. Calcd for $C_{55}H_{35}BrN_6{\cdot}H_2O$: C, 75.25; H, 4.25; N, 9.57; found: C, 75.46; H, 4.38; N, 9.68.

5-[4-(Naphthalen-2-yl)pyrrolo[1,2-*a*]quinoxalin-7-yl]-10,15,20-triphenylporphyrin (4f).

Purple solid; yield: (21 mg, 60%); mp >300°C; UV λ_{max} ($\varepsilon\times10^{-4}$, M^{-1} cm^{-1}): 423 (63.00), 517 (3.50), 551 (1.91), 597 (0.34) and 647 (0.97) nm; λ_{Em} ($CHCl_3$; λ_{Ex} 420 nm): 652, 717 nm; IR (film) ν_{max}/cm^{-1}: 3317, 1596, 1473, 1351, 1221, 1157, 971, 799, 753, 729, 700; ^{1}H NMR (400 MHz, $CDCl_3$) δ: 8.98 (1H, d, J = 2.20 Hz, *meso*-ArH), 8.94 (2H, d, J = 5.13 Hz, β-pyrrolic H), 8.87-8.86 (6H, m, β-pyrrolic H), 8.65 (1H, s, ArH), 8.37 (1H, dd, J_1 = 8.05, J_2 = 2.20 Hz, *meso*-ArH), 8.28 (1H, d, J = 1.46 Hz pyrrolic H-1′), 8.26-8.21 (7H, m, ArH and *meso*-ArH), 8.03 (1H, d, J = 8.79 Hz, ArH), 8.02-7.99 (1H, m, ArH), 7.94-7.92 (1H, m, ArH), 7.79-7.75 (10H, m, *meso*-ArH), 7.57-7.55 (2H, m, ArH), 7.28 (1H, d, J = 2.93 Hz, pyrrolic H-3′), 7.09 (1H, dd, J = 2.93, 2.20 Hz, pyrrolic H-2′), -2.72 (2H, s, internal NH); ^{13}C NMR (100 MHz, $CDCl_3$) δ: 155.41, 142.11, 139.39, 135.86, 135.62, 134.97, 134.56, 134.15, 133.73, 133.16, 131.18, 128.79, 128.56, 128.40, 127.79, 127.71, 126.95, 126.88, 126.67, 126.42, 126.04, 125.81, 120.34, 120.26, 118.71, 115.05, 114.55, 111.88, 109.27; ESI-MS: m/z = 831.6 $(M+H)^+$; Anal. Calcd for $C_{59}H_{38}N_6$: C, 85.28; H, 4.61; N, 10.11; found: C, 84.98; H, 4.78; N, 10.24.

5-[4-(Pyren-1-yl)pyrrolo-[1,2-*a*]quinoxalin-7-yl]-10,15,20-triphenylporphyrin (4g).

Purple solid; yield: (28 mg, 71%); mp >300°C; UV λ_{max} ($\varepsilon\times10^{-4}$, M^{-1} cm^{-1}): 422 (59.97), 517 (3.32), 552 (1.82), 596 (0.32) and 648 (0.87) nm; λ_{Em} ($CHCl_3$; λ_{Ex} 420 nm): 652, 717 nm; IR (film) ν_{max}/cm^{-1}: 3318, 1596, 1474, 1352, 1155, 1072, 971, 800, 752, 729, 701; ^{1}H NMR (400

MHz, $CDCl_3$) δ: 9.02 (1H, d, *J* = 2.20 Hz, *meso*-ArH), 9.00 (2H, d, *J* = 4.39 Hz, *β*-pyrrolic H), 8.89 (2H, d, *J* = 5.13 Hz, *β*-pyrrolic H), 8.86 (4H, s, *β*-pyrrolic H), 8.49-8.43 (3H, m, ArH), 8.35 (1H, d, *J* = 8.05 Hz, ArH), 8.32-8.29 (2H, m, *meso*-ArH and pyrrolic H-1′), 8.24-8.22 (6H, m, *meso*-ArH), 8.19 (1H, d, *J* = 8.05 Hz, ArH), 8.16 (2H, s, ArH), 8.08 (1H, d, *J* = 9.52 Hz, ArH), 8.02 (1H, t, *J* = 7.32 Hz, ArH), 7.79-7.74 (10H, m, *meso*-ArH), 7.01 (1H, dd, *J* = 2.93, 2.20 Hz, pyrrolic H-2′), 6.74 (1H, d, *J* = 3.66 Hz, pyrrolic H-3′), -2.71 (2H, s, internal NH); ^{13}C NMR (100 MHz, $CDCl_3$) δ: 156.26, 142.12, 139.48, 135.70, 134.98, 134.56, 134.01, 132.60, 132.07, 131.26, 130.92, 129.46, 128.23, 127.99, 127.71, 127.47, 127.35, 127.15, 127.01, 126.67, 126.53, 126.09, 125.48, 125.37, 125.18, 125.12, 124.67, 124.60, 120.36, 120.27, 118.67, 114.98, 114.60, 111.99, 109.76; ESI-MS: m/z = 905.6 $(M+H)^+$; Anal. Calcd for $C_{65}H_{40}N_6$: C, 86.26; H, 4.45; N, 9.29; found: C, 85.96; H, 4.53; N, 9.36.

5-[4-(9*H*-Fluoren-2-yl)pyrrolo[1,2-*a*]quinoxalin-7-yl]-10,15,20-triphenyl-porphyrin (4h).

Purple solid; yield: (27 mg, 72%); mp >300°C; UV λ_{max} ($\varepsilon\times10^{-4}$, M^{-1} cm^{-1}): 423 (73.28), 517 (3.90), 552 (2.07), 596 (0.38) and 648 (0.96) nm; λ_{Em} ($CHCl_3$; λ_{Ex} 420 nm): 652, 717 nm; IR (film) ν_{max}/cm^{-1}: 3318, 1592, 1474, 1352, 1219, 1072, 971, 800, 752, 735, 701; ^1H NMR (400 MHz, $CDCl_3$) δ: 8.96 (1H, d, *J* = 2.20 Hz, *meso*-ArH), 8.93 (2H, d, *J* = 5.13 Hz, *β*-pyrrolic H), 8.86-8.85 (6H, m, *β*-pyrrolic H), 8.35 (1H, dd, J_1 = 8.05, J_2 = 2.20 Hz, *meso*-ArH), 8.32 (1H, s, ArH), 8.27-8.22 (7H, m, pyrrolic H-1′ and *meso*-ArH), 8.18 (1H, d, *J* = 8.05 Hz, ArH), 7.96 (1H, d, *J* = 8.05 Hz, ArH), 7.87 (1H, d, *J* = 7.32 Hz, ArH), 7.76-7.75 (10H, m, *meso*-ArH), 7.58 (1H, d, *J* = 7.32 Hz, ArH), 7.41 (1H, t, *J* = 7.32 Hz, ArH), 7.34 (1H, t, *J* = 7.32 Hz, ArH), 7.24-7.23 (1H, m, pyrrolic H-3′), 7.09-7.07 (1H, m, pyrrolic H-2′), 4.02 (2H, s, CH_2), -2.72 (2H, s, internal NH), ^{13}C NMR (100 MHz, $CDCl_3$) δ: 155.70, 143.91, 143.66, 143.54, 142.11, 141.13, 139.33, 136.93, 135.54, 134.95, 134.56, 133.58, 131.31, 127.71,

127.65, 127.23, 126..86, 126.67, 125.77, 125.36, 125.14, 120.35, 120.26, 119.83, 118.76, 114.99, 114.43, 111.84, 109.26, 37.031; ESI-MS: m/z = 869.5 $(M+H)^+$; Anal. Calcd for $C_{62}H_{40}N_6$: C, 85.69; H, 4.64; N, 9.67; found: C, 85.38; H, 4.53; N, 9.56.

General procedure for the synthesis of zinc(II) pyrrolo-[1,2-*a*]quinoxalino-porphyrins 5 and 6.

To the solution of free-base porphyrin **4g** or **4h** (30 mg) in chloroform (15 mL), a solution of $Zn(OAc)_2 \cdot 2H_2O$ (18 mg, 0.081 mmol) in methanol (1.5 mL) was added and the reaction mixture was stirred at room temperature for 30 min. After completion of the reaction, the mixture was diluted with $CHCl_3$ (20 mL) and washed with water (3 × 30 mL). The organic layer was dried over sodium sulfate and evaporated to dryness under vacuum. The residue obtained was subjected to column chromatography on silica gel using $CHCl_3$ as eluent. The pure product was obtained after eluting the column with 5% methanol in chloroform.

Zinc(II) 5-[4-(pyren-1-yl)pyrrolo[1,2-*a*]quinoxalin-7-yl]-10,15,20-triphenyl-porphyrin (5).

Purple solid; yield: (27 mg, 84%); mp >300°C; UV λ_{max} ($\varepsilon \times 10^{-4}$, M^{-1} cm^{-1}): 425 (104.90), 554 (3.80) and 594 (1.00) nm; λ_{Em} ($CHCl_3$; λ_{Ex} 420 nm): 605, 652 nm; IR (film) ν_{max}/cm^{-1}: 1480, 1339, 1206, 1070, 1003, 955, 796, 753, 736, 701; ^1H NMR (400 MHz, $CDCl_3$) δ: 9.10 (2H, d, *J* = 4.39 Hz, *β*-pyrrolic H), 9.02 (1H, d, *J* = 1.46 Hz, *meso*-ArH), 8.99 (2H, d, *J* = 4.39 Hz, *β*-pyrrolic H), 8.95 (4H, s, *β*-pyrrolic H), 8.49-8.44 (3H, m, ArH), 8.34 (1H, d, *J* = 8.05 Hz, ArH), 8.32-8.27 (2H, m, *meso*-ArH and pyrrolic H-1′), 8.25-8.20 (6H, m, *meso*-ArH), 8.17 (1H, d, *J* = 7.32 Hz, ArH), 8.15 (2H, s, ArH), 8.06 (1H, d, *J* = 9.52 Hz, ArH), 8.02-7.98 (1H, m, ArH), 7.78-7.73 (10H, m, *meso*-ArH), 6.99 (1H, dd, *J* = 2.93, 2.20 Hz, pyrrolic H-2′), 6.72 (1H, d, *J* = 4.39 Hz, pyrrolic H-3′); ^{13}C NMR (100 MHz, $CDCl_3$) δ: 156.13, 150.28, 150.25, 142.77, 140.16, 135.55, 134.90, 134.48, 134.41, 133.96, 132.60, 132.22, 132.04, 132.01, 131.94, 131.24, 130.88, 129.44, 128.20, 127.95, 127.48, 127.32, 127.03, 126.88, 126.59,

126.51, 126.02, 125.41, 125.32, 125.20, 125.12, 124.64, 124.56, 121.31, 121.24, 119.64, 114.85, 114.51, 111.82, 109.66; ESI-MS: m/z = 967.5 $(M+H)^+$; Anal. Calcd for $C_{65}H_{38}N_6Zn{\cdot}H_2O$: C, 79.14; H, 4.09; N, 8.52; found: C, 78.96; H, 3.96; N, 8.34.

Zinc(II) 5-[4-(9*H*-fluoren-2-yl)pyrrolo[1,2-*a*]quinoxalin-7-yl]-10,15,20-triphenyl-porphyrin (6).

Purple solid; Yield: (28 mg, 87%); mp >300°C; UV λ_{max} ($\varepsilon \times 10^{-4}$, M^{-1} cm^{-1}): 425 (117.50), 553 (4.20) and 594 (1.20) nm; λ_{Em} ($CHCl_3$; λ_{Ex} 420 nm): 606, 654 nm; IR (film) ν_{max}/cm^{-1}: 1475, 1339, 1206, 1070, 1003, 796, 753, 718, 700; ^1H NMR (400 MHz, $CDCl_3$) δ: 9.03 (2H, d, *J* = 4.39 Hz, *β*-pyrrolic H), 8.96 (6H, s, *β*-pyrrolic H), 8.95-8.94 (1H, m, *meso*-ArH), 8.35 (1H, dd, J_1 = 8.05, J_2 = 2.20 Hz, *meso*-ArH), 8.28-8.22 (7H, m, pyrrolic H-1′ and *meso*-ArH), 8.19 (1H, d, *J* = 8.05 Hz, ArH), 8.11 (1H, d, *J* = 8.05 Hz, ArH), 7.89 (1H, d, *J* = 8.05 Hz, ArH), 7.82 (1H, d, *J* = 7.32 Hz, ArH), 7.79-7.74 (10H, m, *meso*-ArH), 7.55 (1H, d, *J* = 7.32 Hz, ArH), 7.39 (1H, t, *J* = 7.32 Hz, ArH), 7.32 (1H, t, *J* = 7.32 Hz, ArH), 7.14 (1H, d, *J* = 4.39 Hz, pyrrolic H-3′), 6.95 (1H, dd, *J* = 2.93, 2.20 Hz, pyrrolic H-2′), 3.98 (2H, s, CH_2) ppm; ^{13}C NMR (100 MHz, $CDCl_3$) δ: 155.59, 150.26, 150.21, 143.89, 143.61, 143.47, 142.76, 141.14, 140.01, 136.93, 135.38, 134.86, 134.45, 134.41, 133.52, 132.13, 132.02, 131.93, 127.62, 127.47, 127.20, 126.79, 126.72, 126.57, 126.49, 125.74, 125.34, 125.12, 121.28, 121.21, 120.33, 119.78, 114.90, 114.35, 111.70, 109.15, 37.01 ppm; ESI-MS: m/z = 931.5 $(M+H)^+$; Anal. Calcd for $C_{62}H_{38}N_6Zn{\cdot}H_2O$: C, 78.35; H, 4.24; N, 8.84; found: C, 78.52; H, 4.34; N, 8.92.
