# Supplementary material for: First synthesis of meso-substituted pyrrolo[1,2-a]quinoxalinoporphyrins
Source: Beilstein J Org Chem. 2014 Apr 8;10:808–13. doi: 10.3762/bjoc.10.76 (PMC4001320; doi:10.3762/bjoc.10.76)
Supplement: File 2 — 1H and 13C NMR spectra of newly synthesized compounds. [file Beilstein_J_Org_Chem-10-808-s002.pdf]

Supporting Information File 2

for

First synthesis of *meso*-substituted pyrrolo[1,2-*a*]quinoxalinoporphyrins

Dileep Kumar Singh and Mahendra Nath*

Address: Department of Chemistry, University of Delhi, Delhi 110 007, India

Email: Mahendra Nath* - mnath@chemistry.du.ac.in

*Corresponding author

^{1}H and ^{13}C NMR spectra of newly synthesized compounds

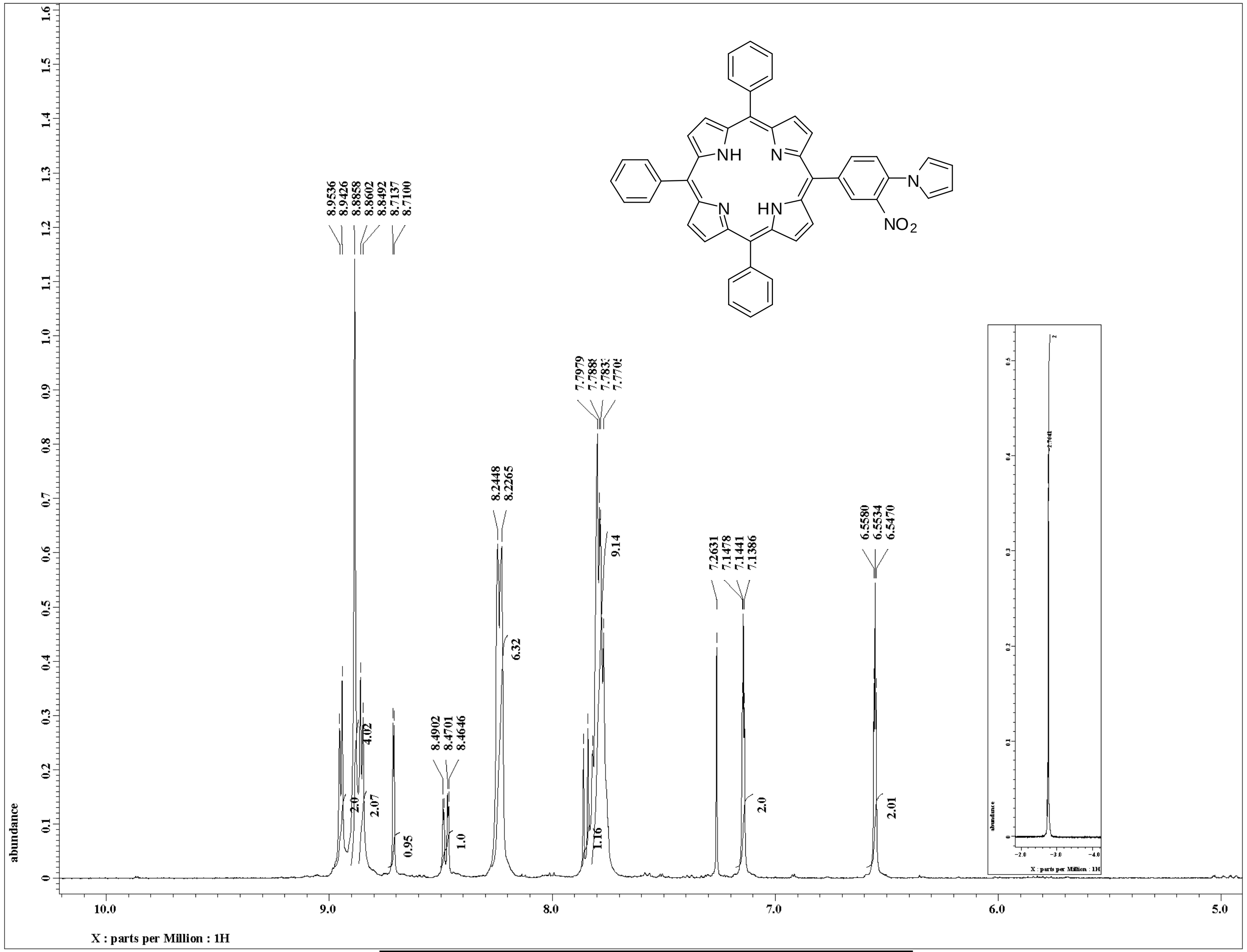

Figure 1: ^{1}H NMR spectrum of compound **2**.

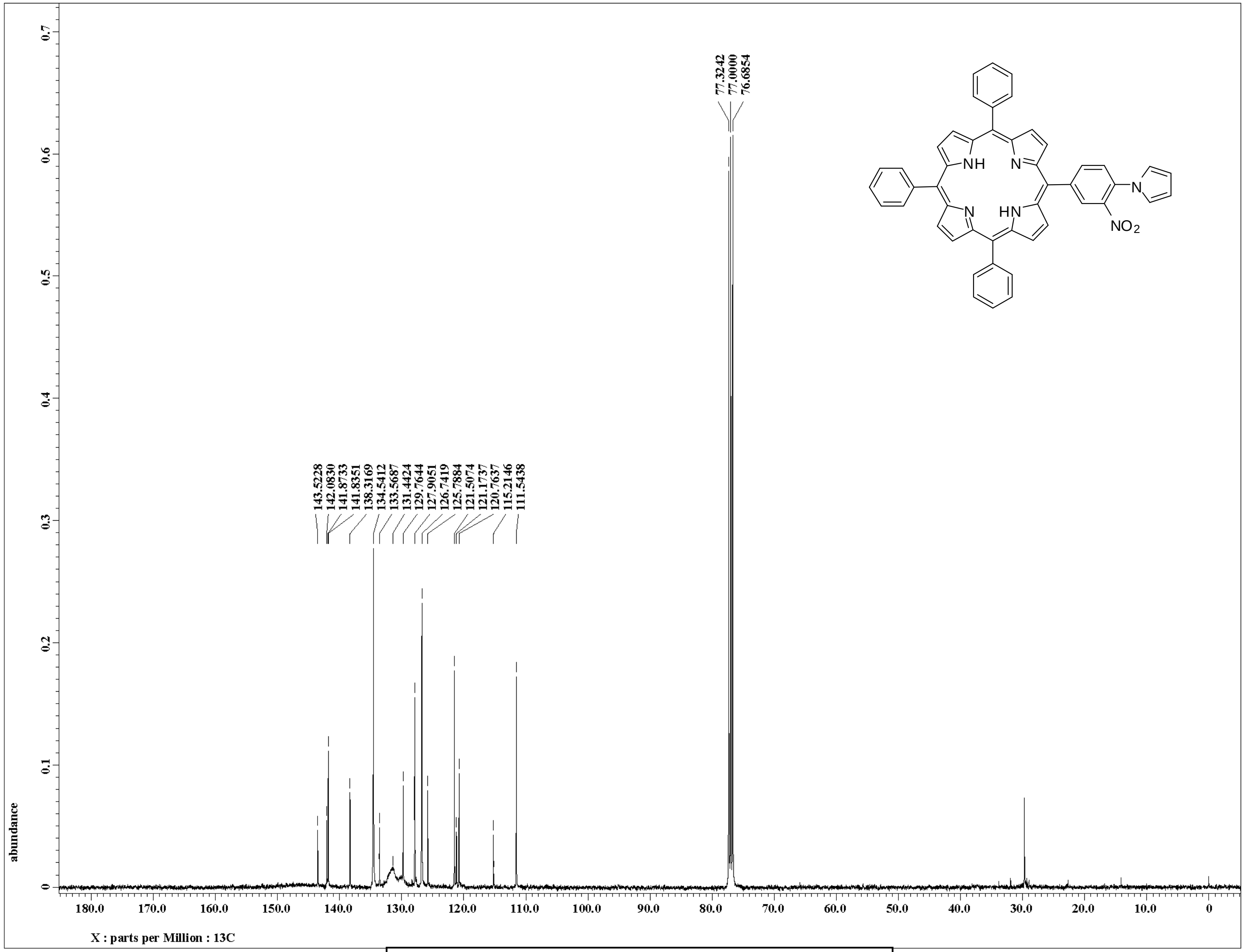

Figure 2: ^{13}C NMR spectrum of compound **2**.

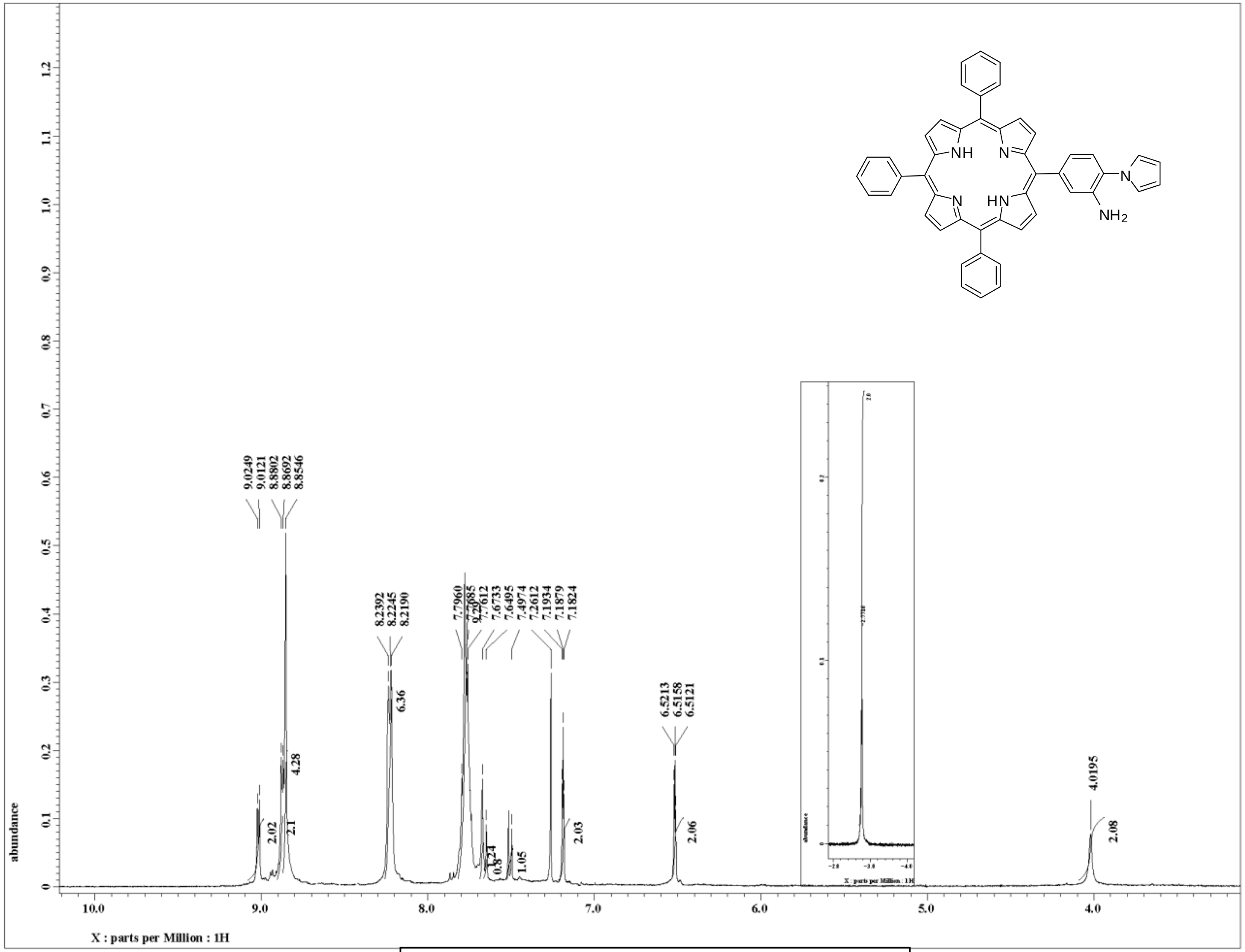

Figure 3: ^{1}H NMR spectrum of compound **3**.

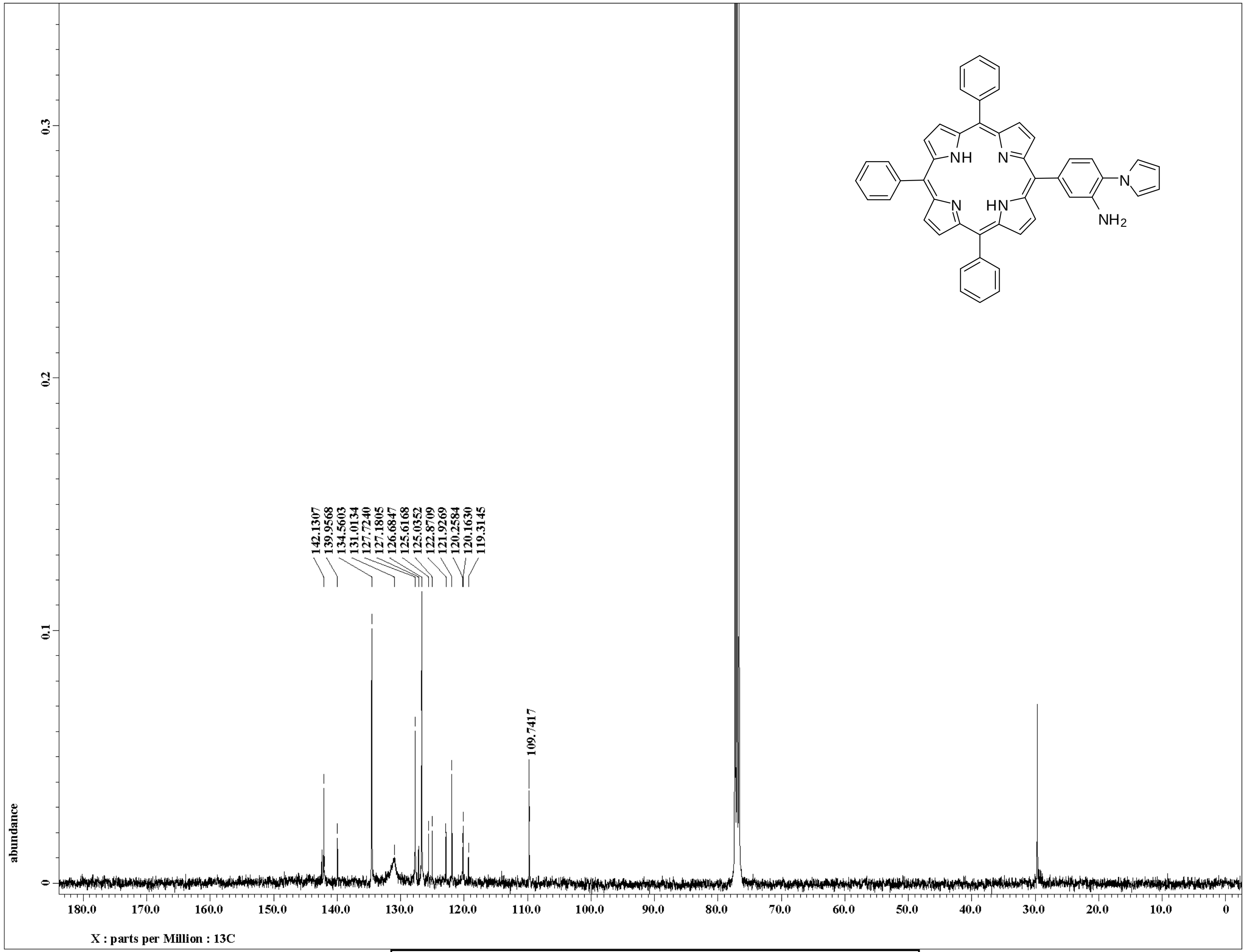

Figure 4: ^{13}C NMR spectrum of compound **3**.

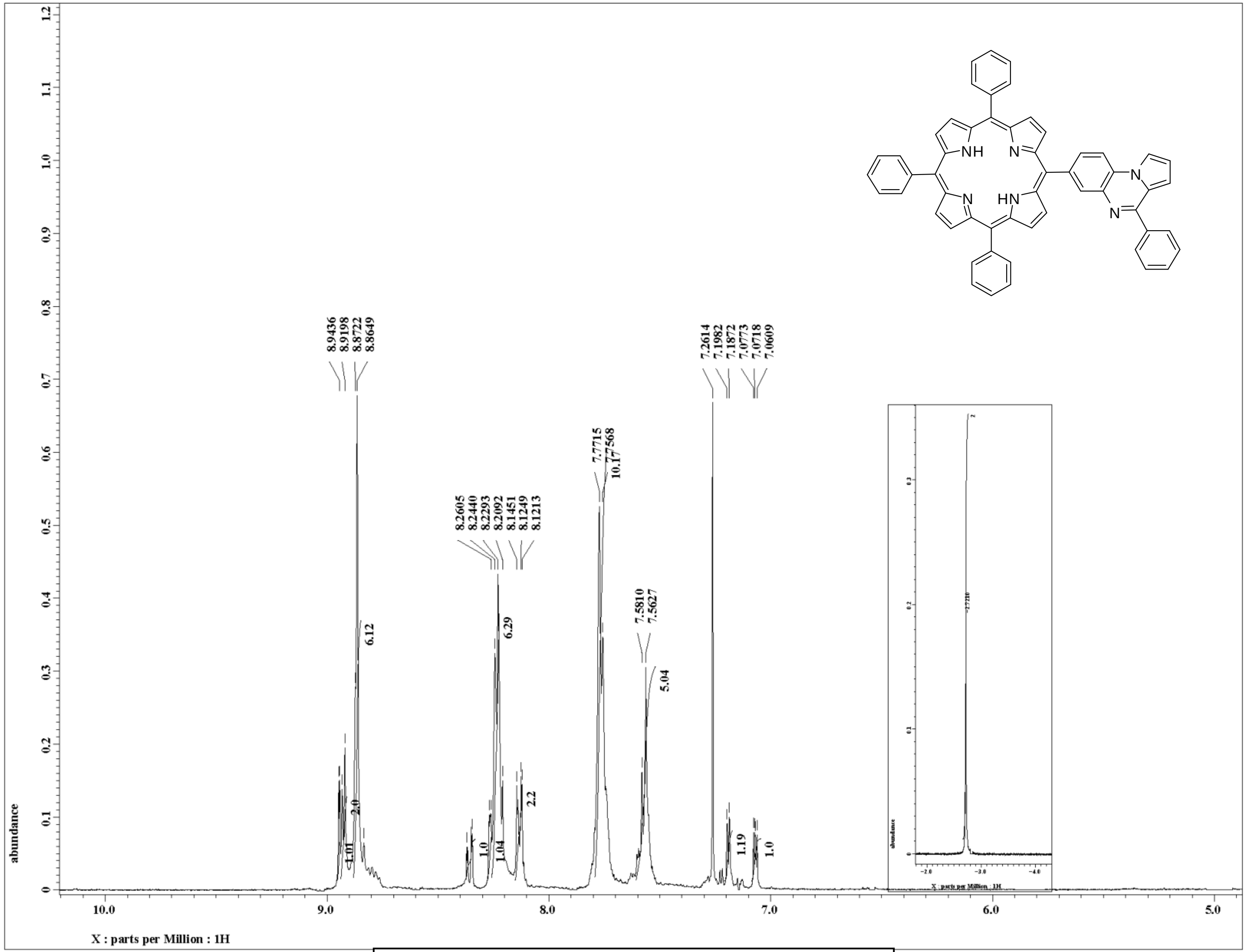

Figure 5: ^{1}H NMR spectrum of compound **4a**.

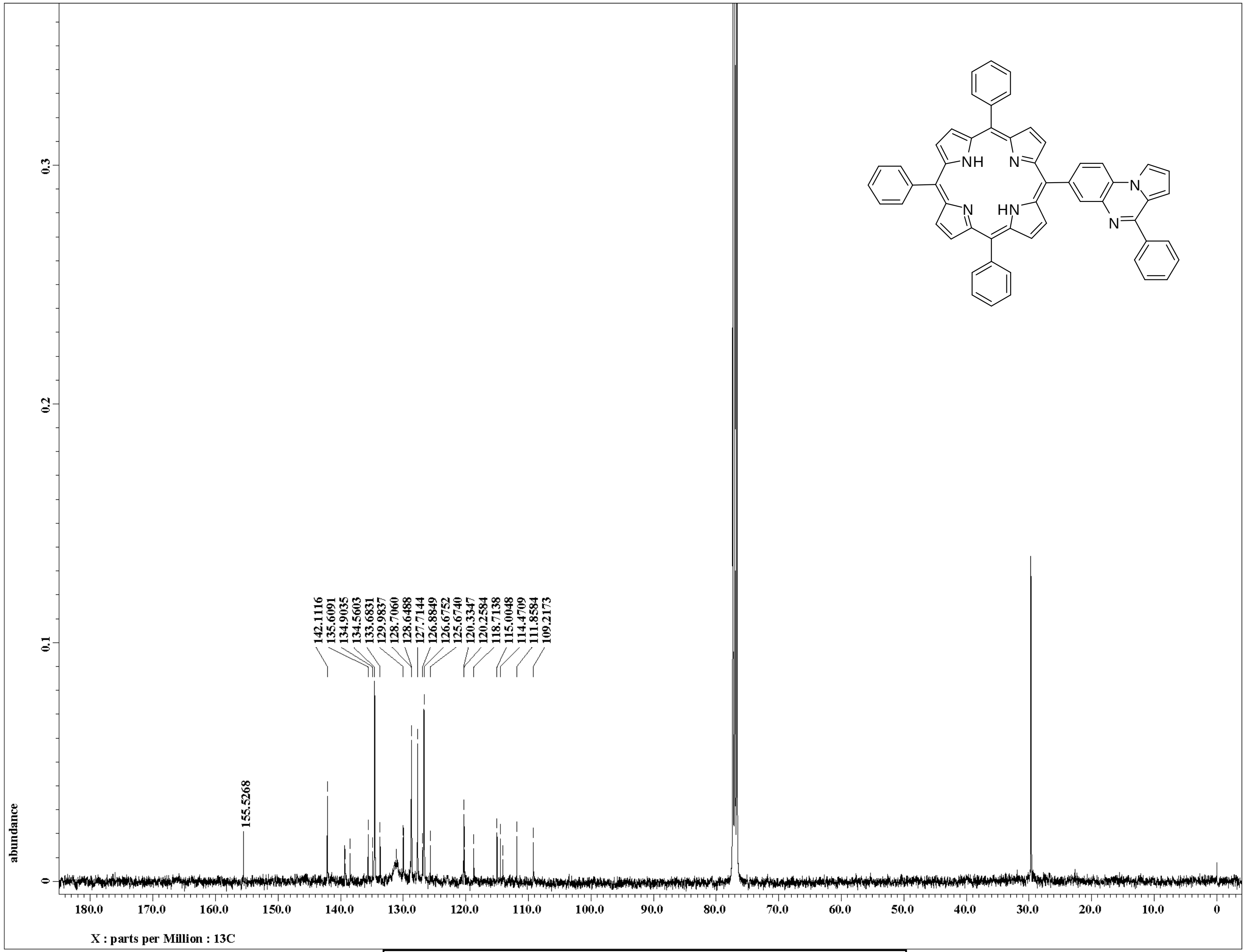

Figure 6: ^{13}C NMR spectrum of compound **4a**.

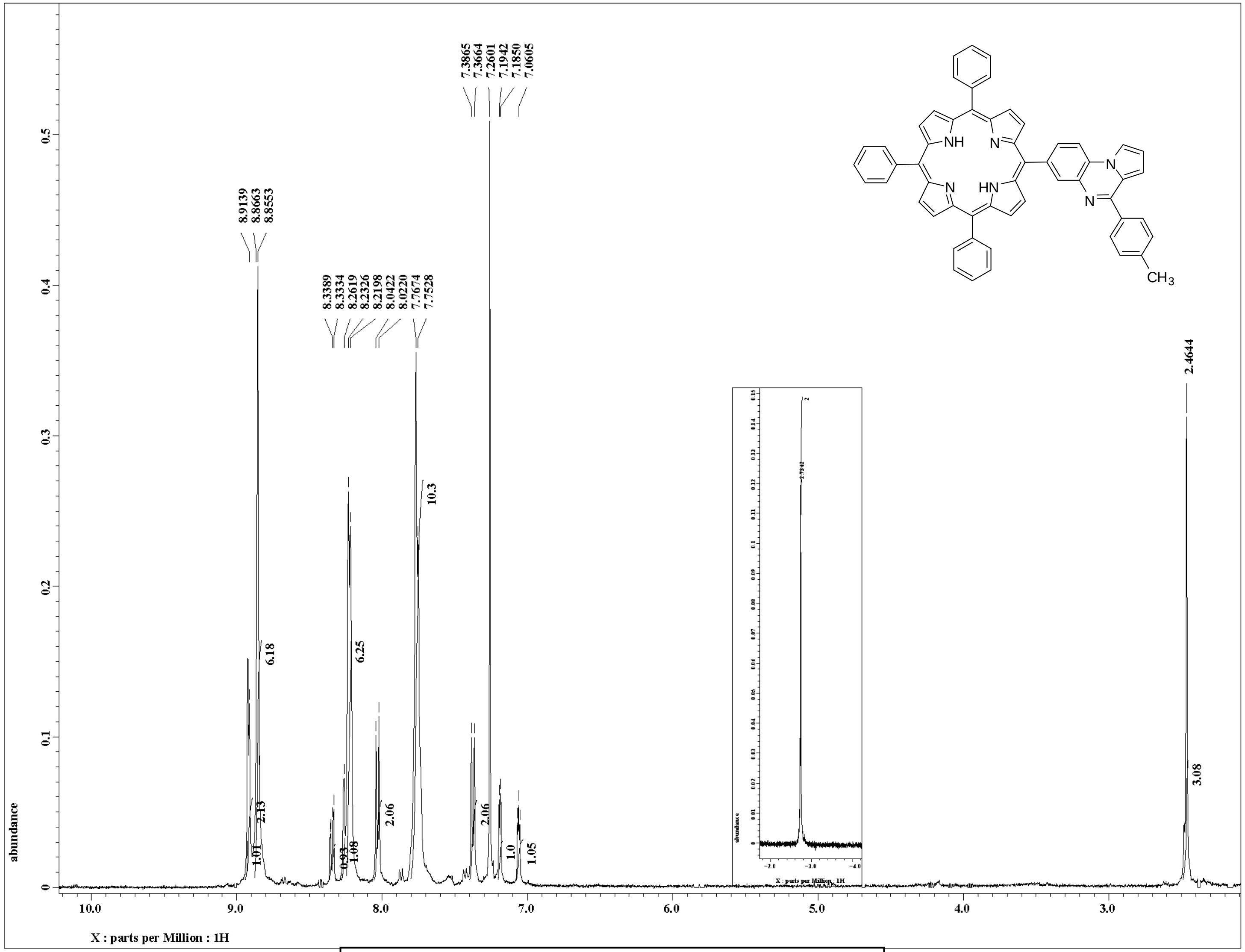

Figure 7: ^{1}H NMR spectrum of compound **4b**.

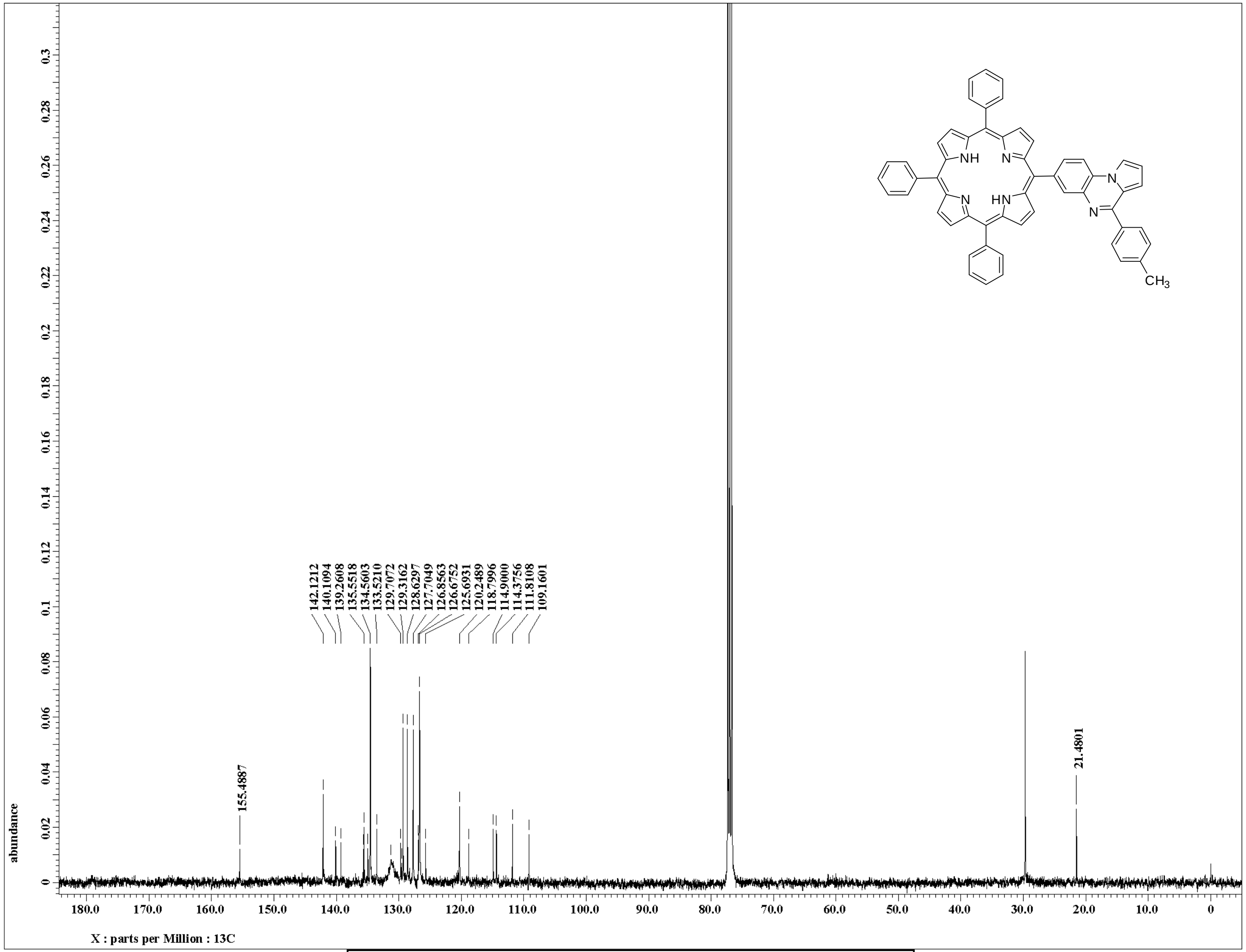

Figure 8: ^{13}C NMR spectrum of compound **4b**.

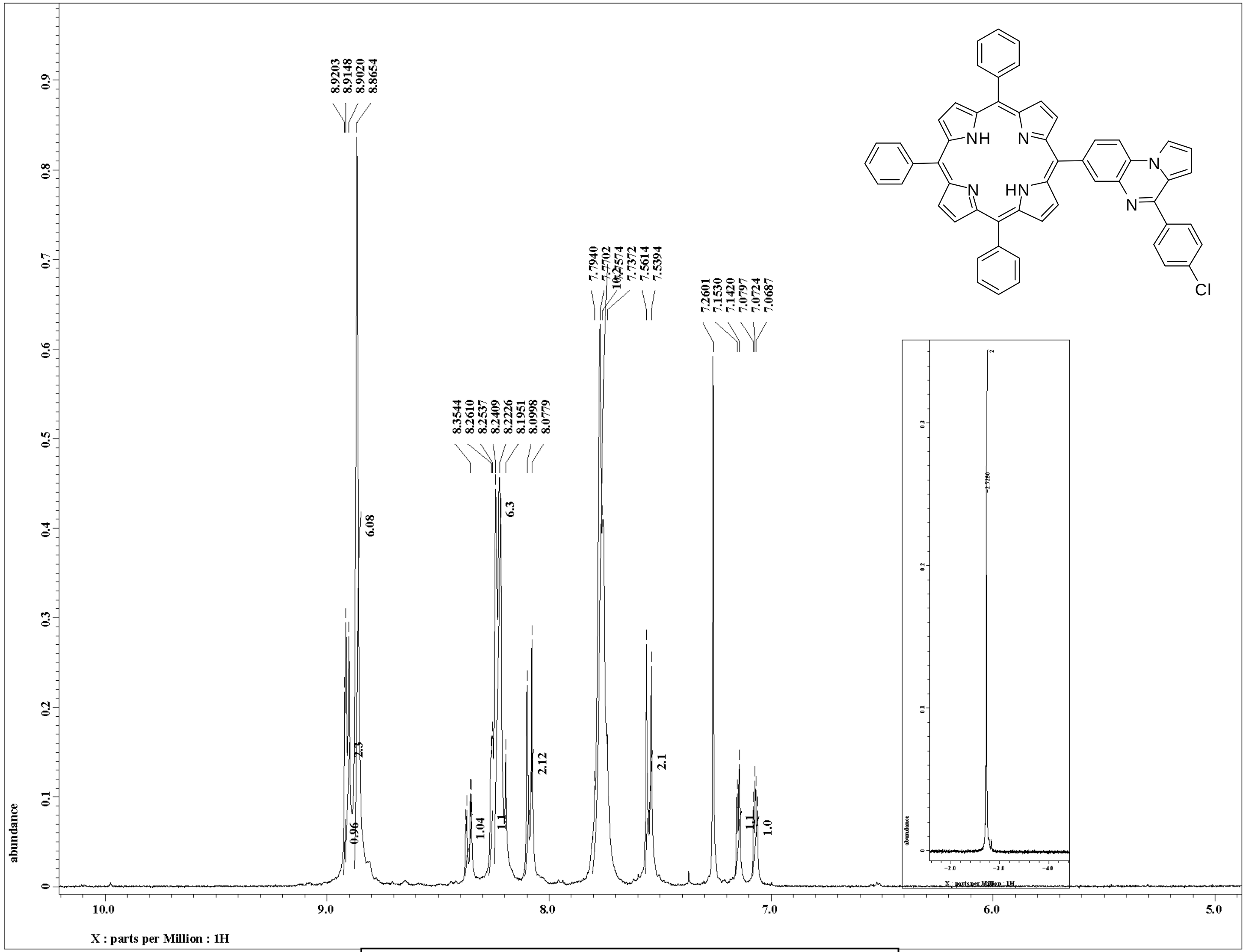

Figure 9: ^{1}H NMR spectrum of compound **4c**.

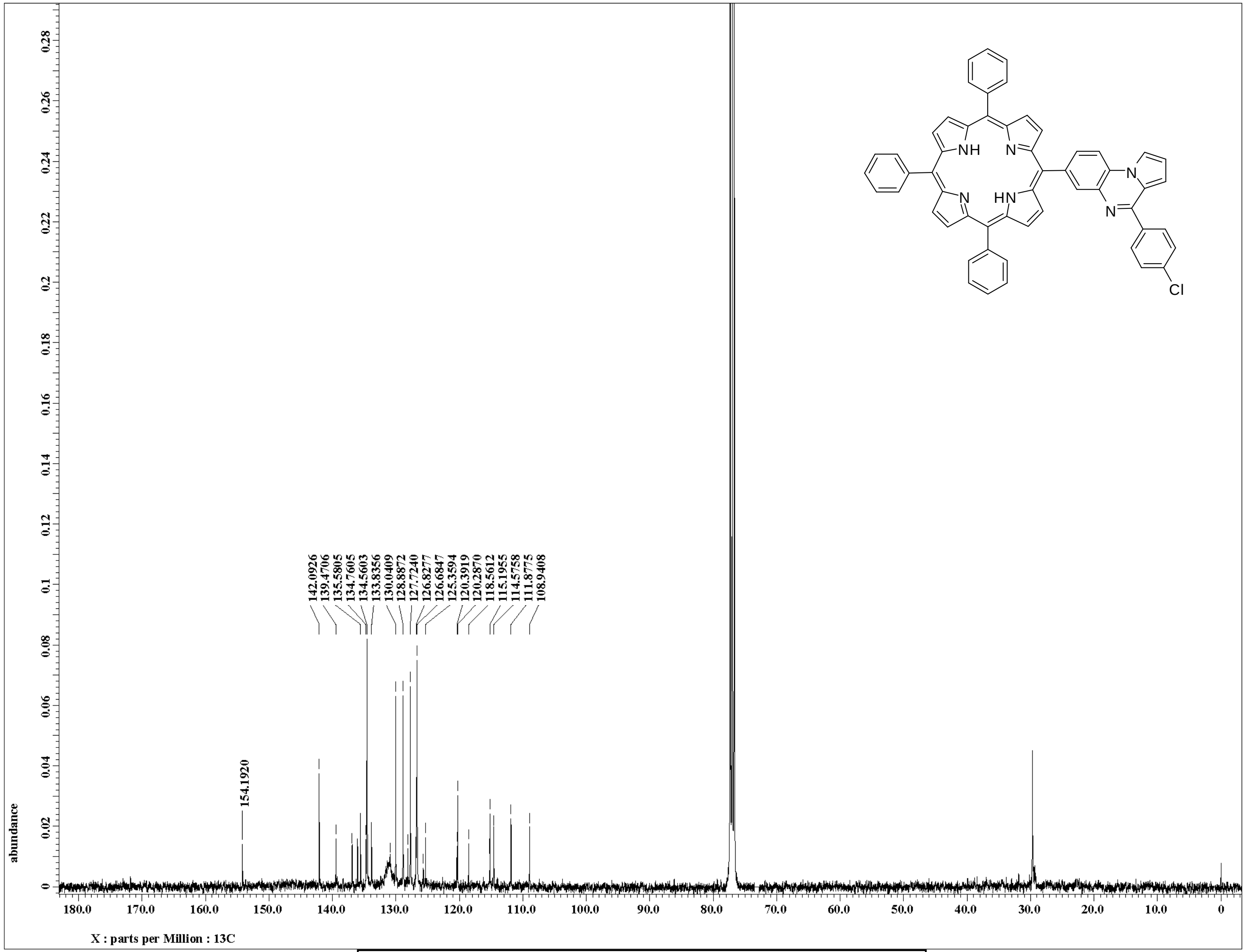

Figure 10: ^{13}C NMR spectrum of compound **4c**.

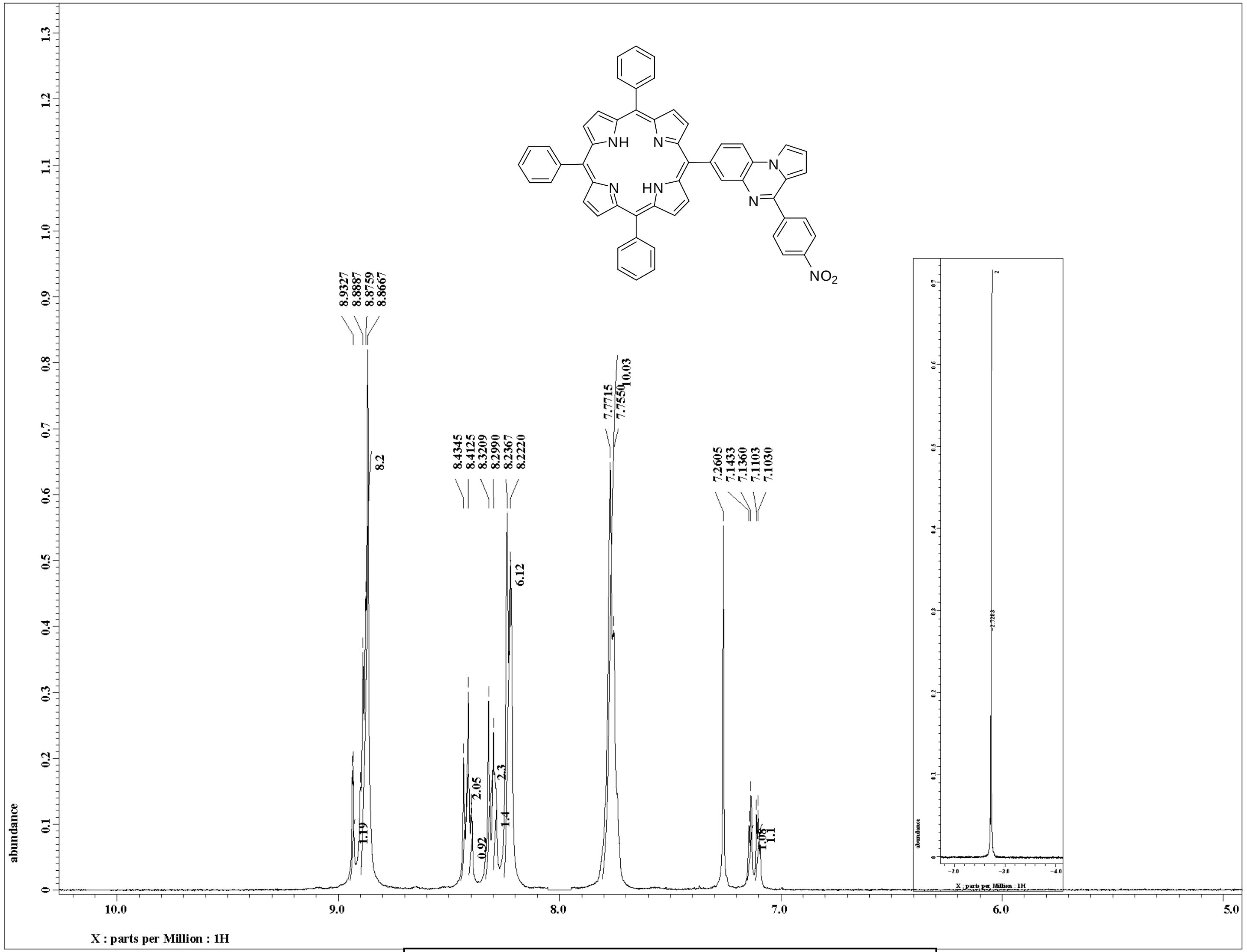

Figure 11: ^{1}H NMR spectrum of compound **4d**.

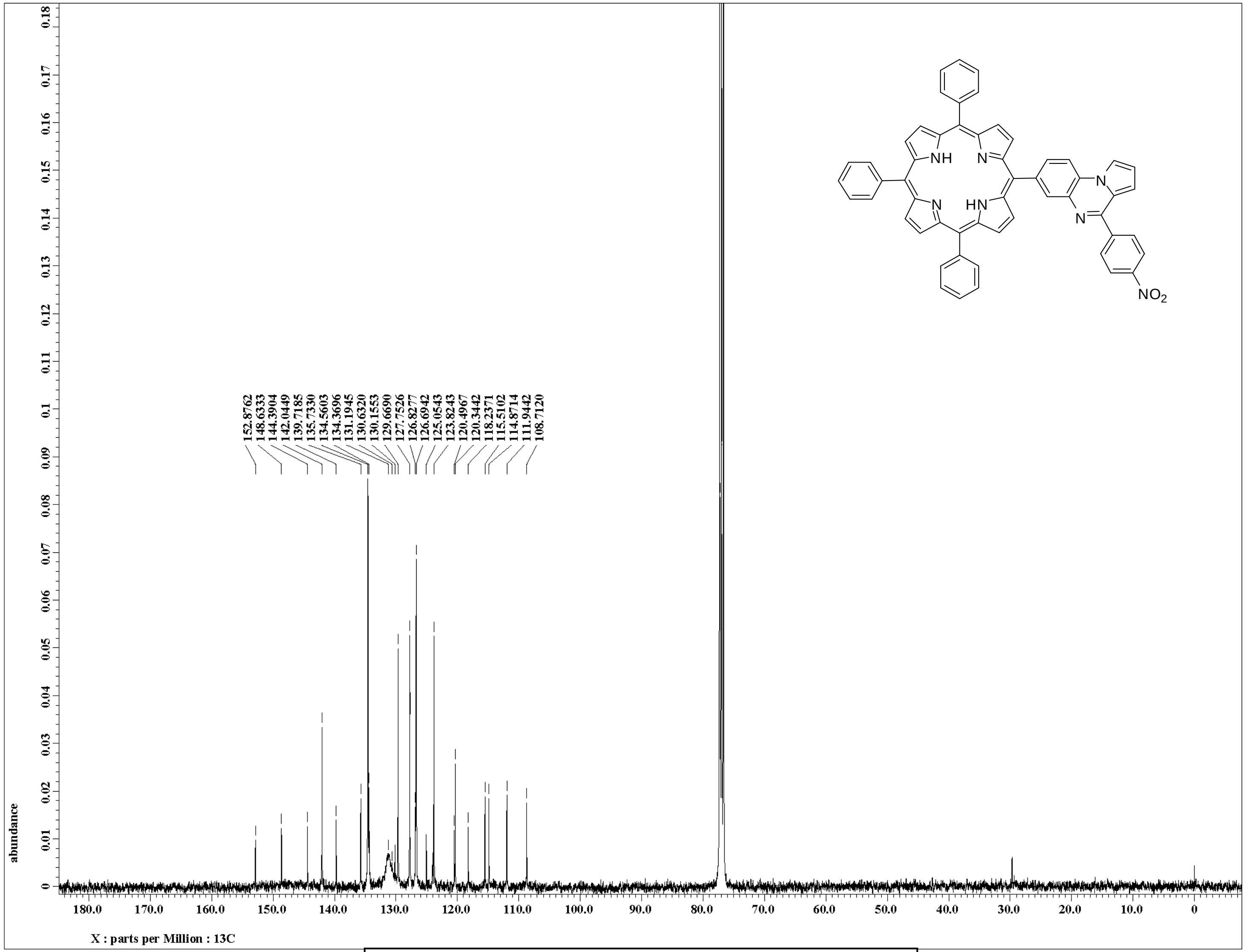

Figure 12: ^{13}C NMR spectrum of compound **4d**.

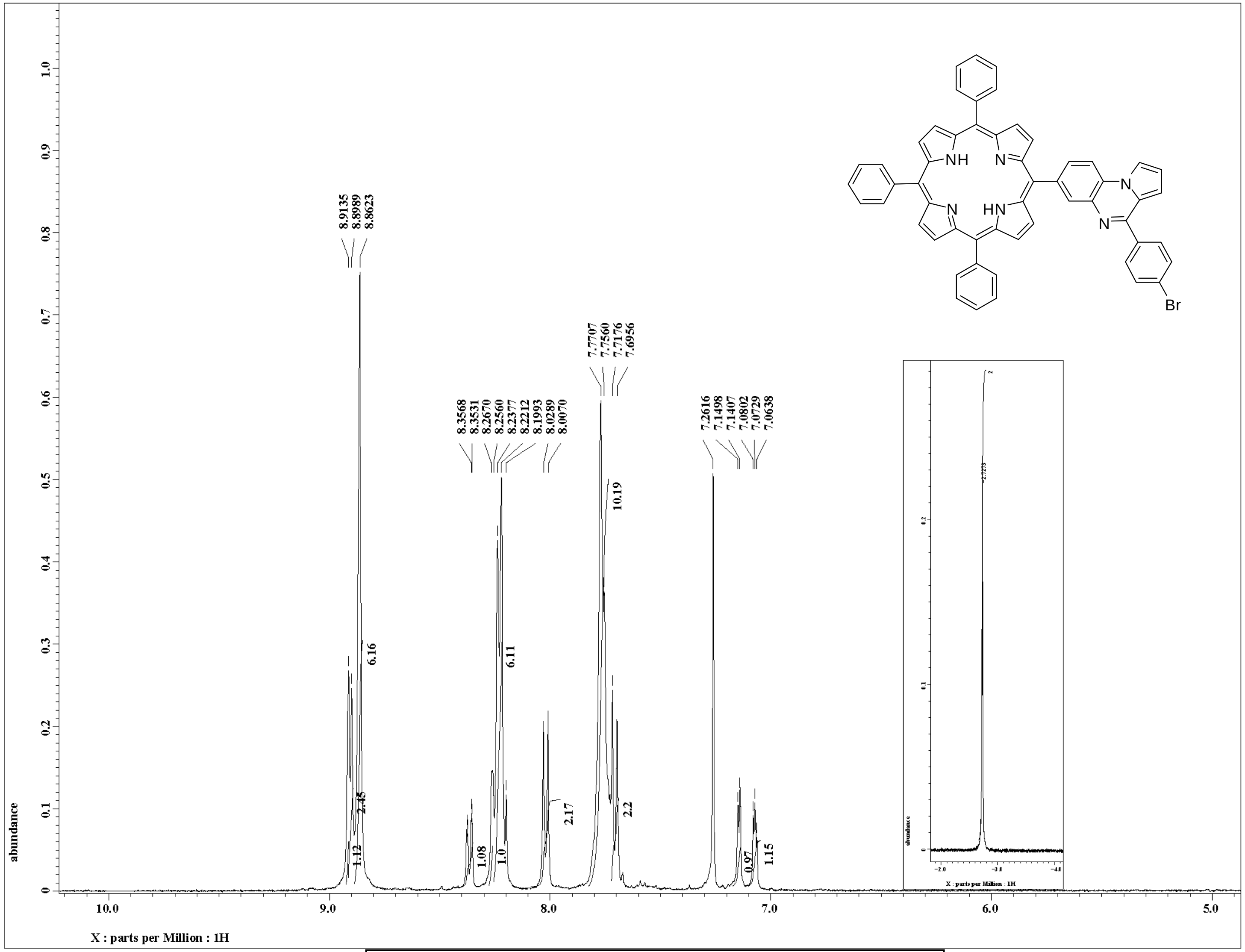

Figure 13: ^{1}H NMR spectrum of compound **4e**.

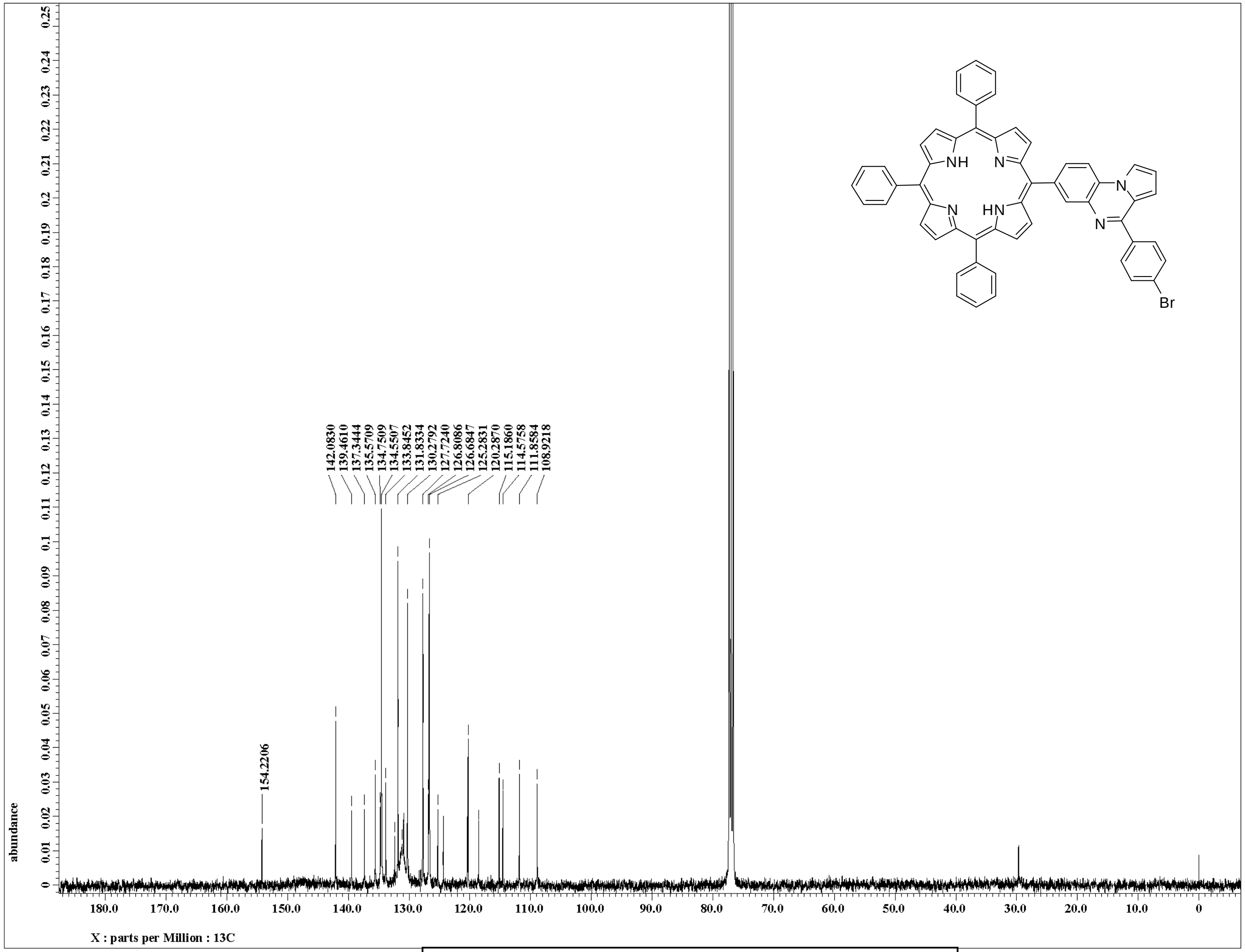

Figure 14: ^{13}C NMR spectrum of compound **4e**.

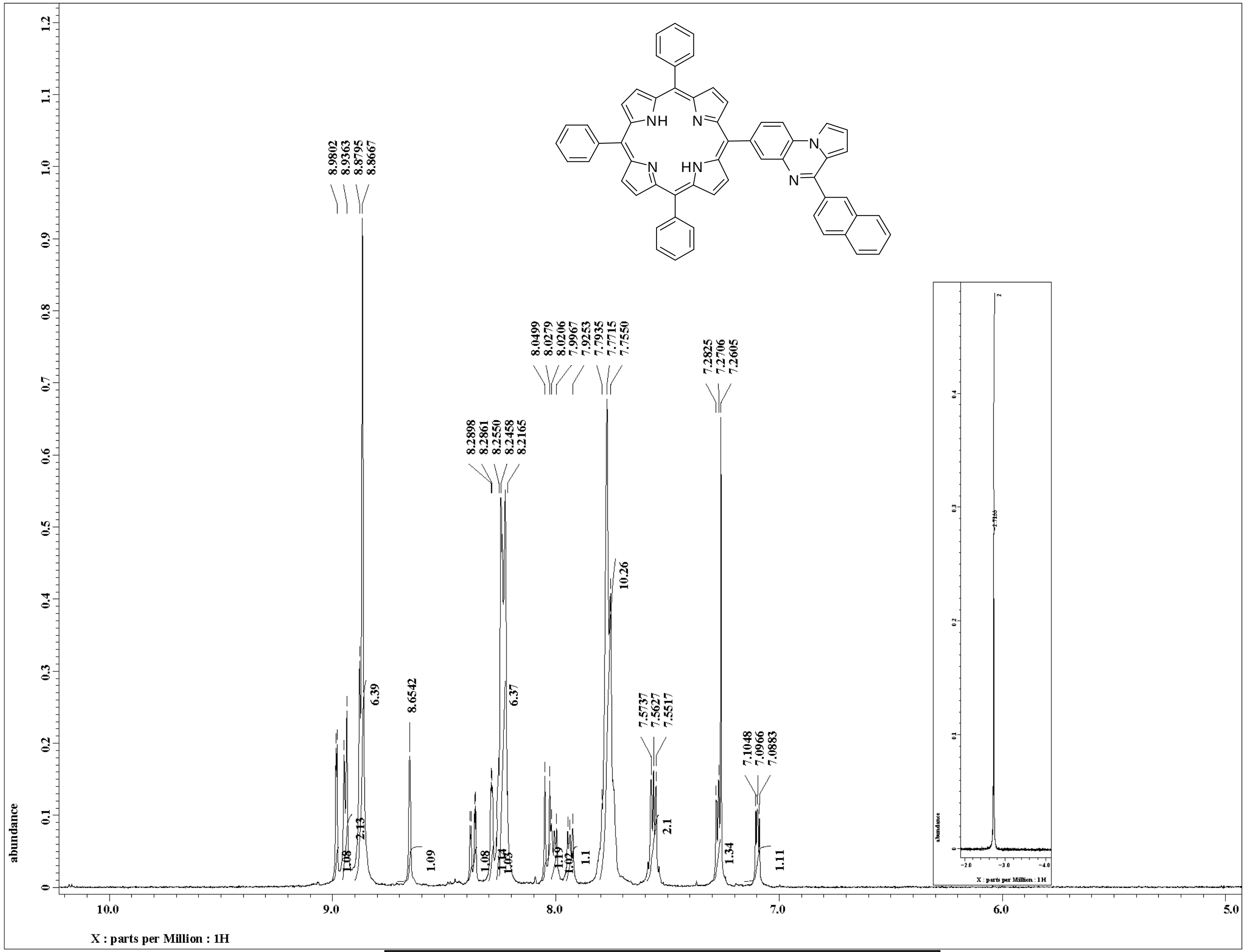

Figure 15: ^{1}H NMR spectrum of compound **4f**.

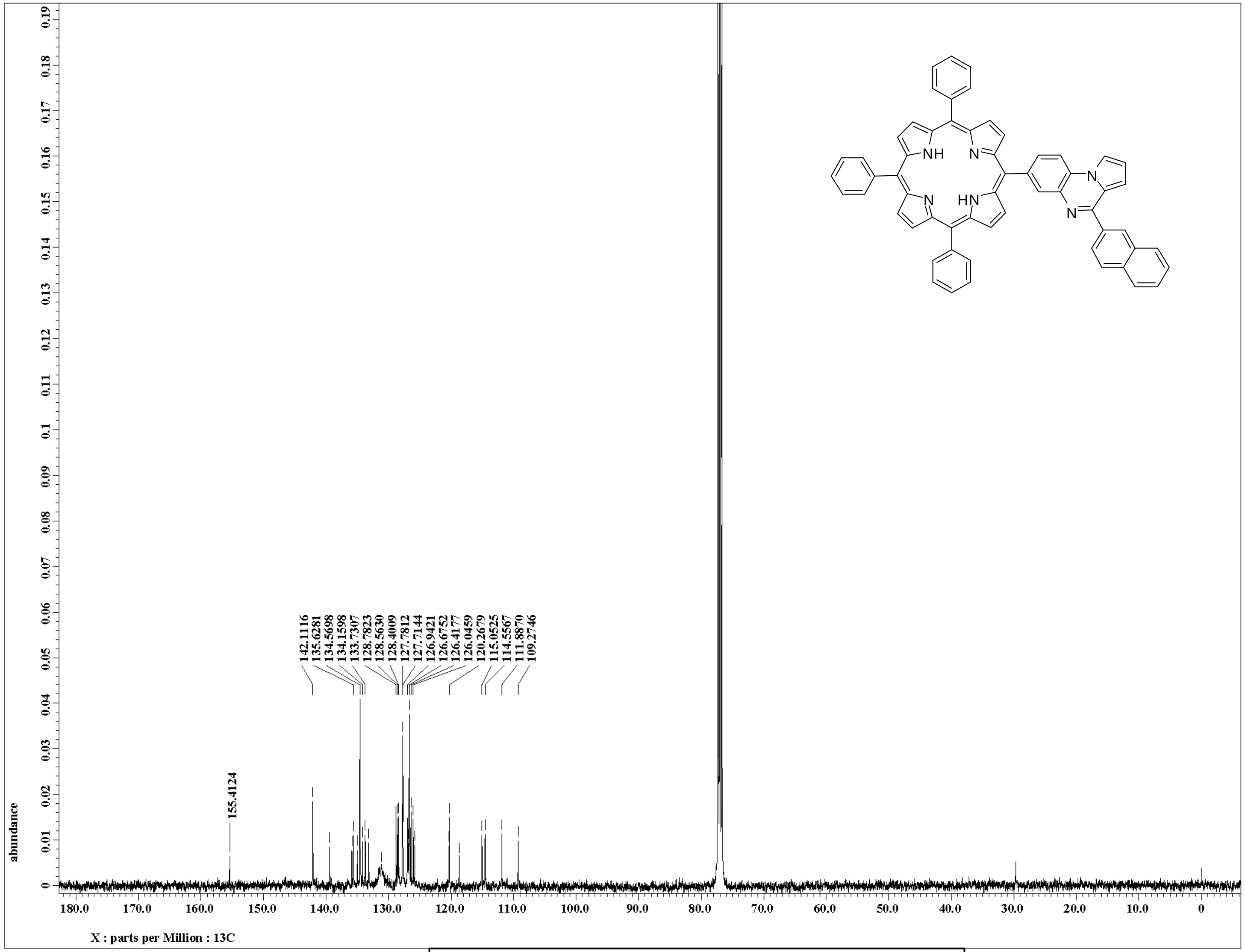

Figure 16: ^{13}C NMR spectrum of compound **4f**.

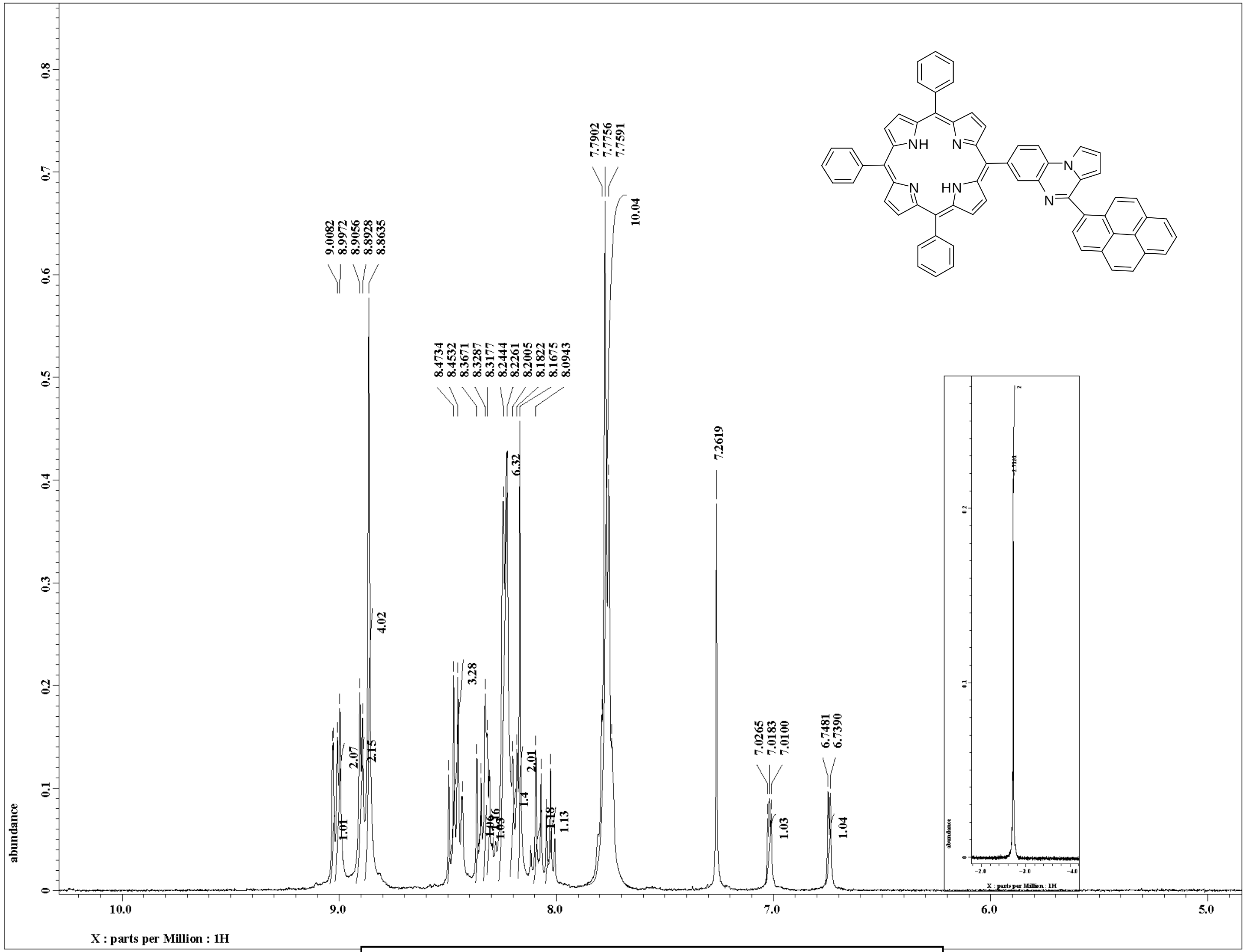

Figure 17: ^{1}H NMR spectrum of compound **4g**.

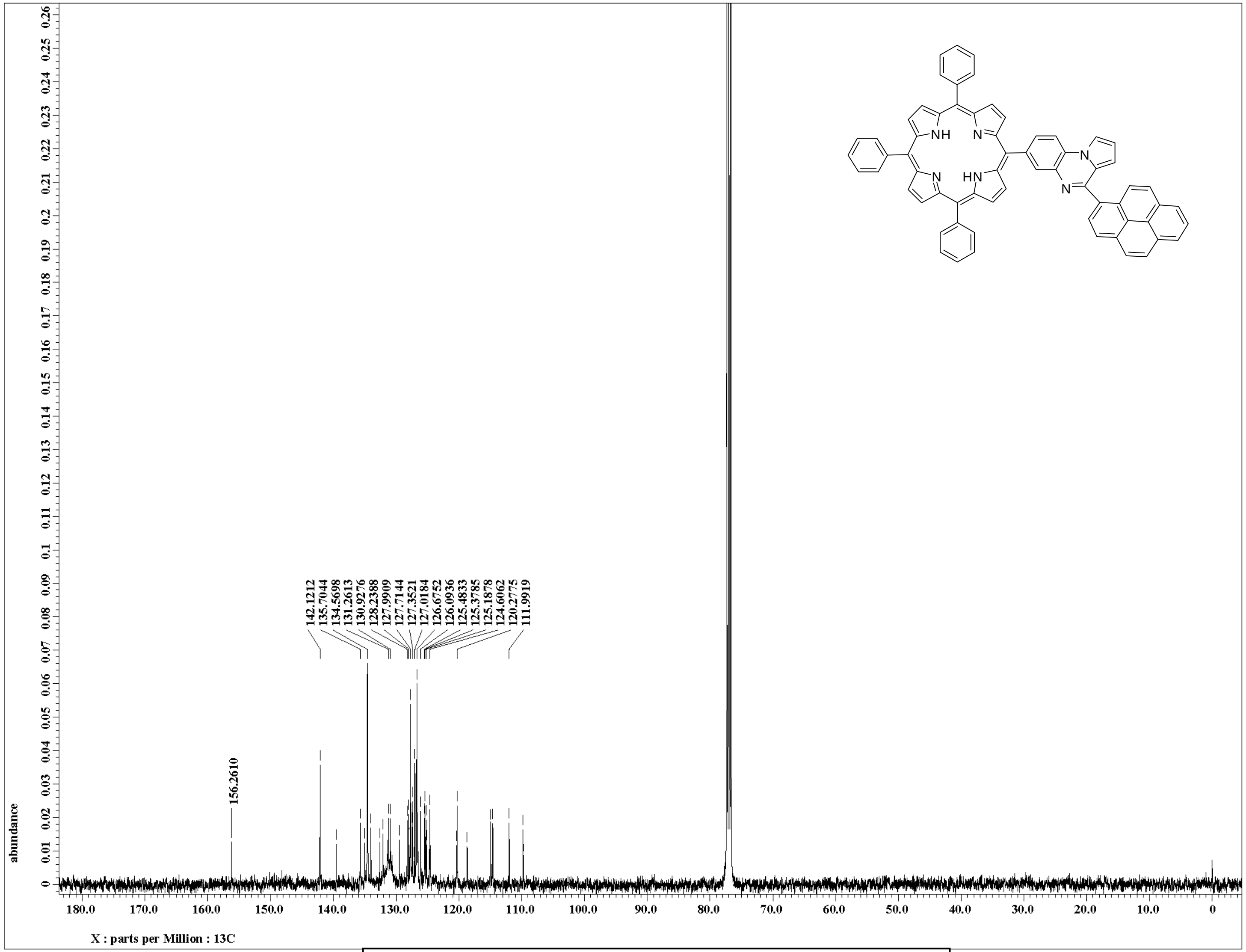

Figure 18: ^{13}C NMR spectrum of compound **4g**.

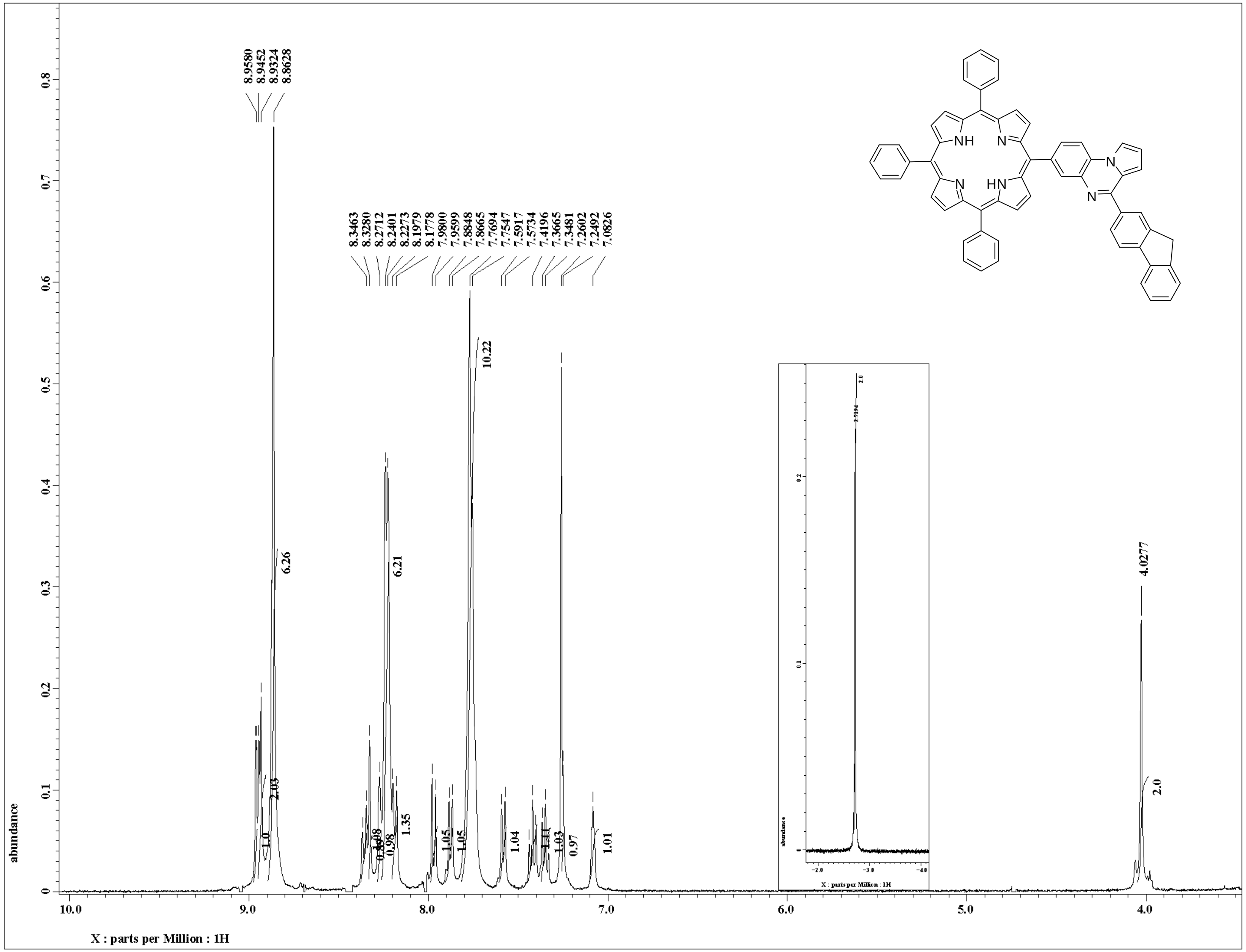

Figure 19: ^{1}H NMR spectrum of compound **4h**.

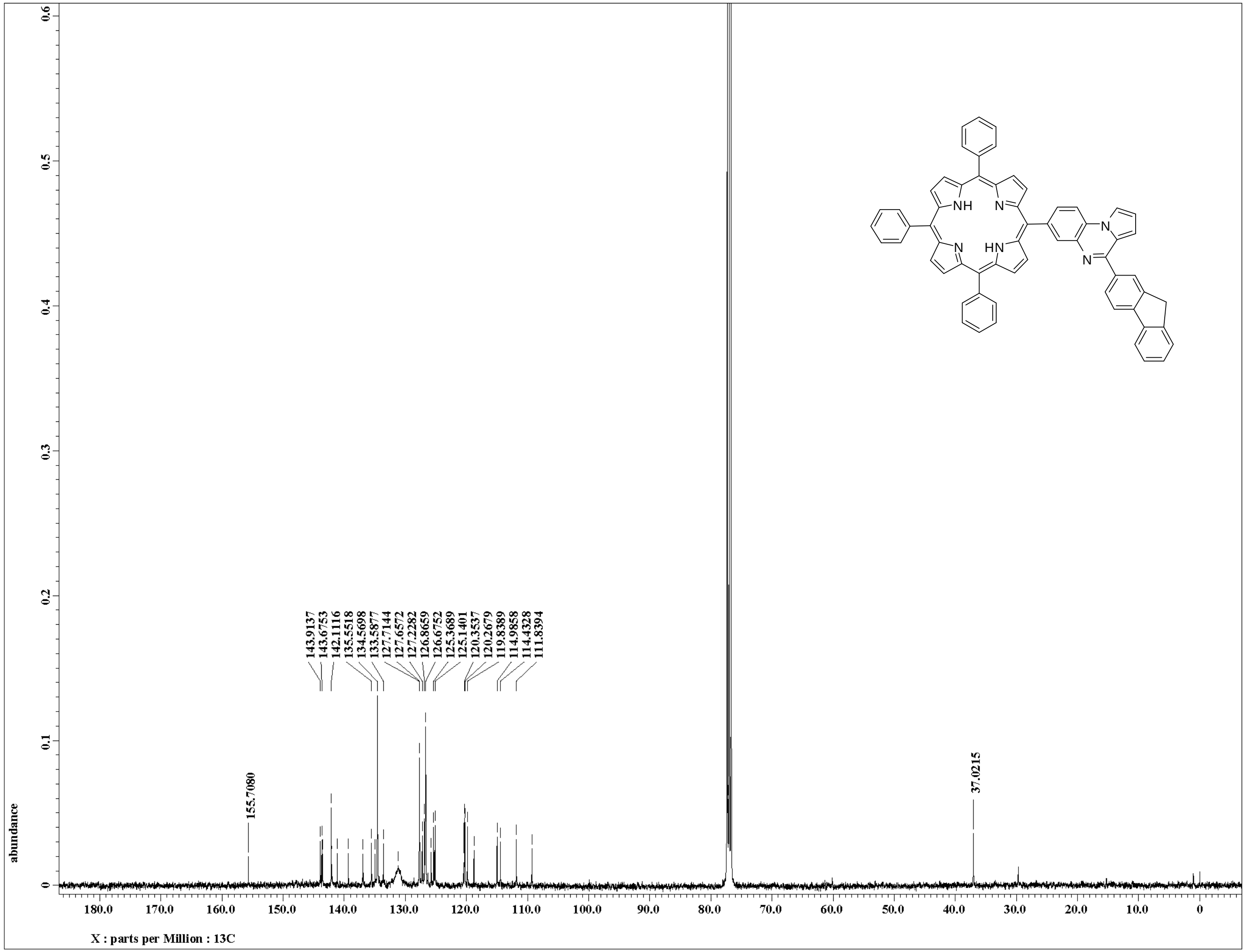

Figure 20: ^{13}C NMR spectrum of compound **4h**.

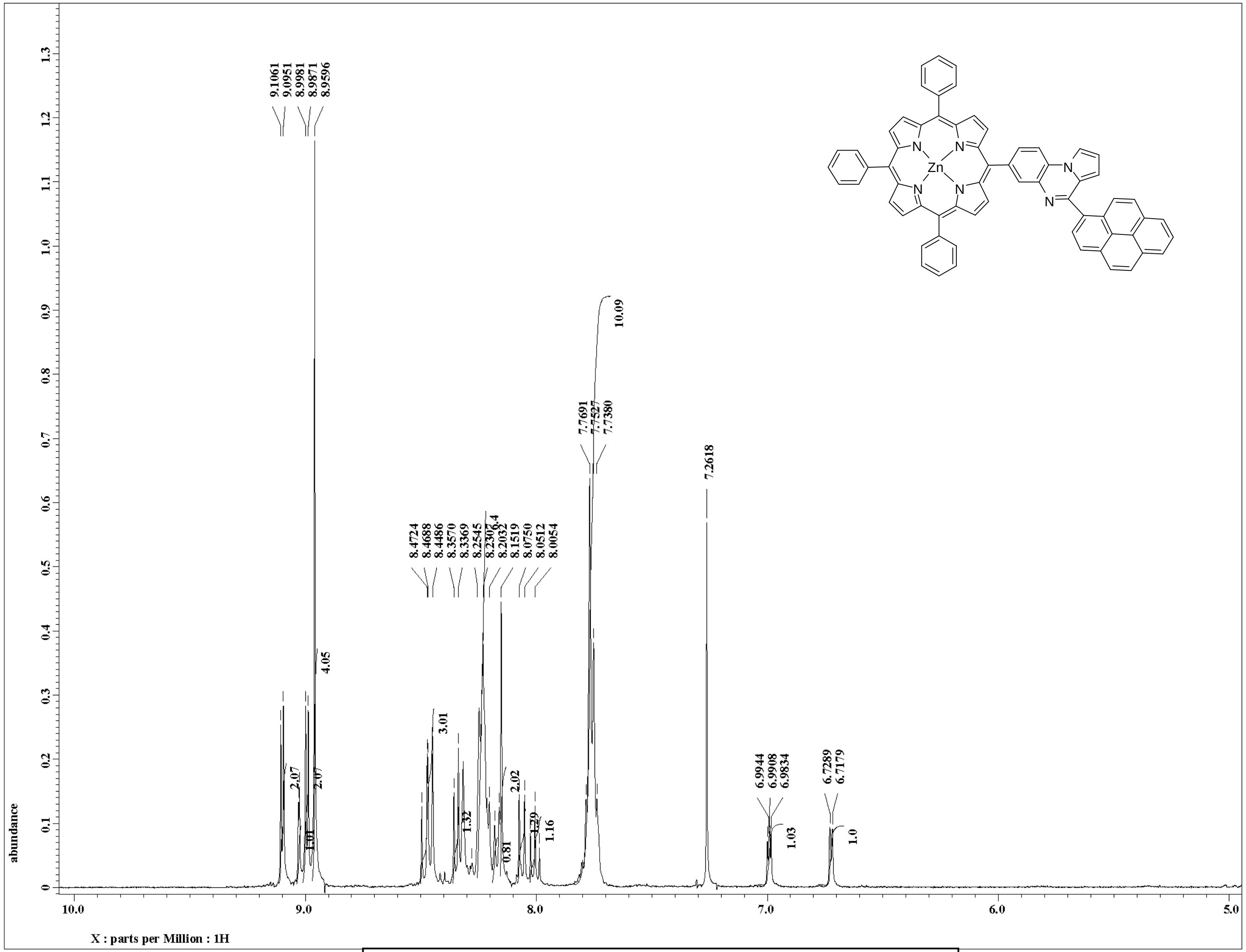

Figure 21: ^{1}H NMR spectrum of compound **5**.

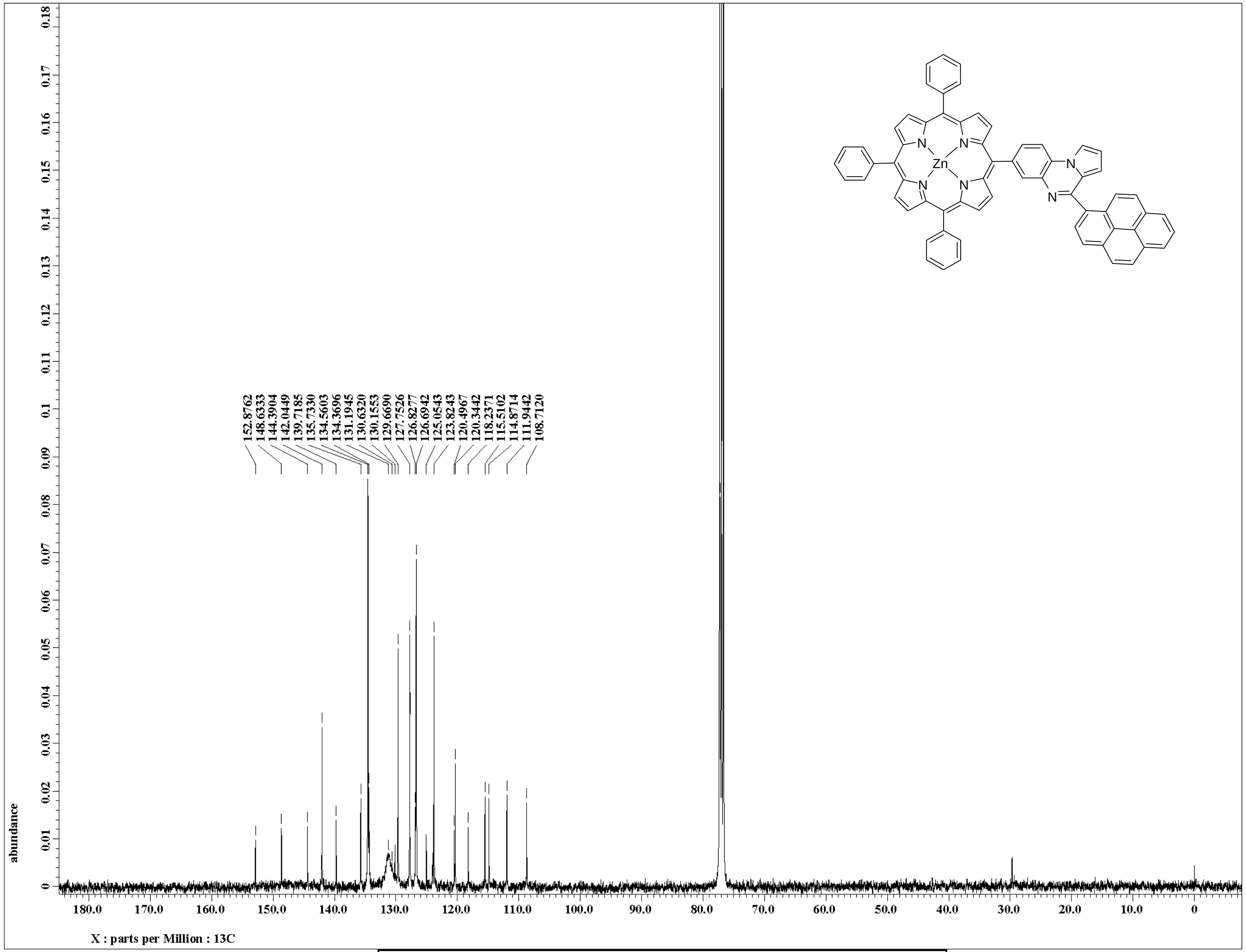

Figure 22: ^{13}C NMR spectrum of compound **5**.

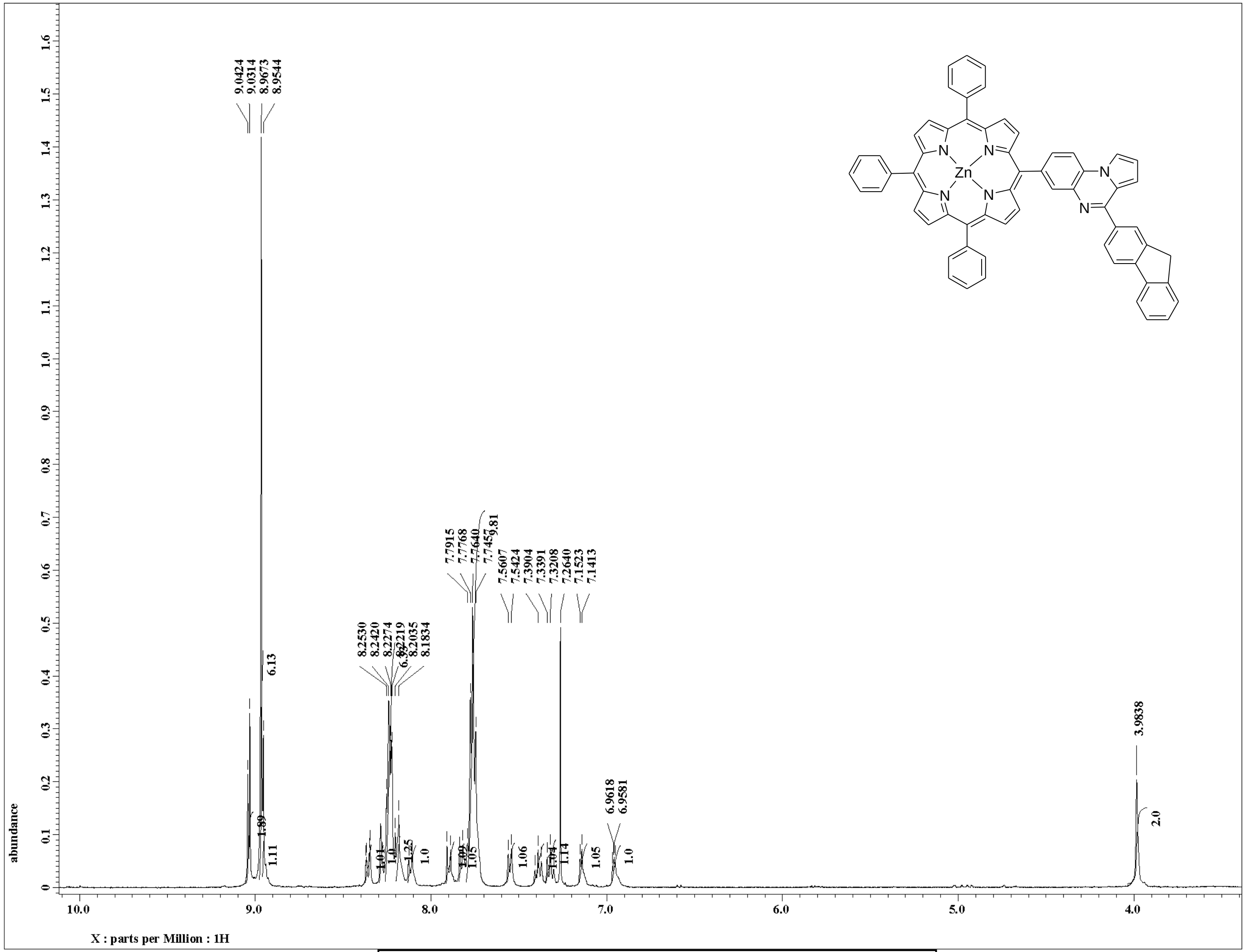

Figure 23: ^{1}H NMR spectrum of compound **6**.

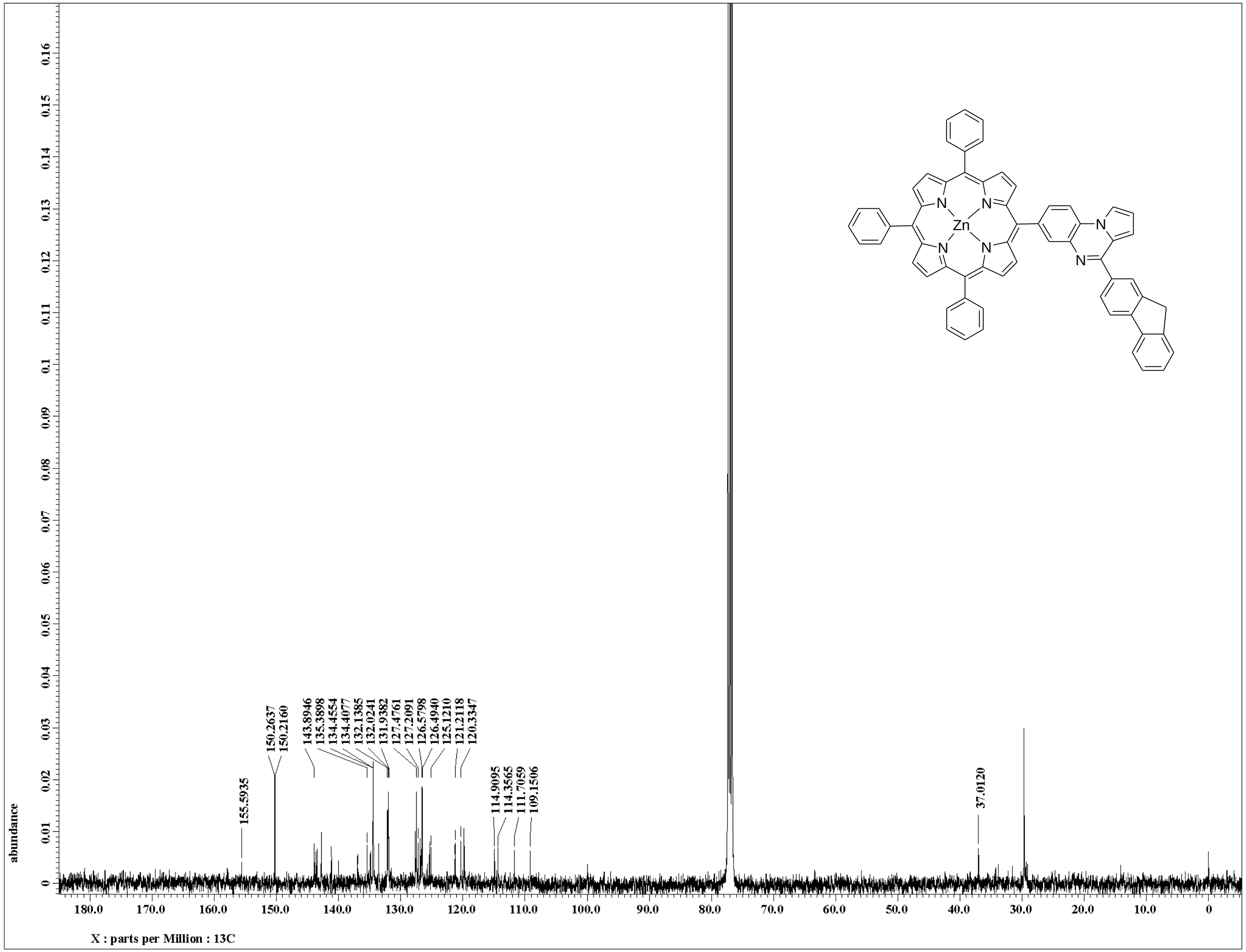

Figure 24: ^{13}C NMR spectrum of compound **6**.
